# Hormetic Effect of Pyroligneous Acids on Conjugative Transfer of Plasmid-mediated Multi-antibiotic Resistance Genes within Bacterial Genus

**DOI:** 10.1021/acsenvironau.2c00056

**Published:** 2022-12-22

**Authors:** Mengying Shao, Liuqingqing Liu, Bingjie Liu, Hao Zheng, Wei Meng, Yifan Liu, Xiao Zhang, Xiaohan Ma, Cuizhu Sun, Xianxiang Luo, Fengmin Li, Baoshan Xing

**Affiliations:** † Institute of Coastal Environmental Pollution Control, Ministry of Education Key Laboratory of Marine Environment and Ecology, College of Environmental Science and Engineering, Frontiers Science Center for Deep Ocean Multispheres and Earth System, Ocean University of China, Qingdao 266100, China; ‡ Marine Ecology and Environmental Science Laboratory, Qingdao National Laboratory for Marine Science and Technology, Qingdao 266071, China; § Ministry of Ecology and Environment, South China Institute of Environmental Sciences, Guangzhou 510535, China; ∥ Sanya Oceanographic Institution, Ocean University of China, Sanya 572000, China; ⊥ Stockbridge School of Agriculture, University of Massachusetts, Amherst, Massachusetts 01003, United States

**Keywords:** antibiotic resistance genes, horizontal gene transfer, soil amendment, pyroligneous acids, oxidative stress response

## Abstract

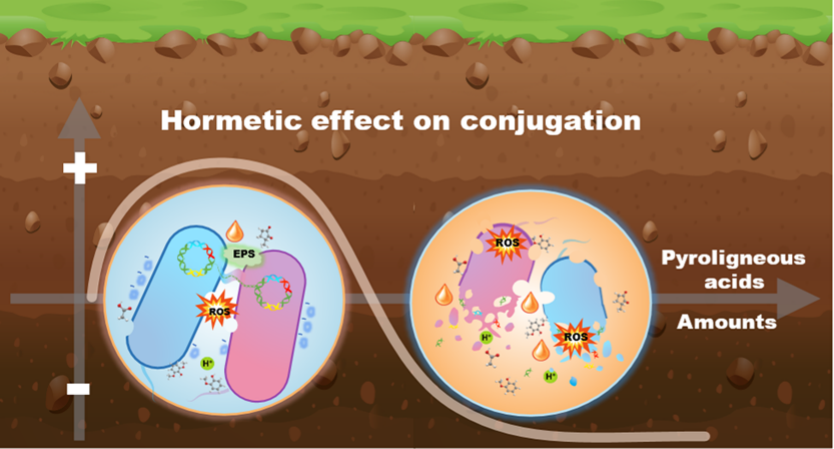

Spread of antibiotic resistance genes (ARGs) by conjugation poses great challenges to public health. Application of pyroligneous acids (PA) as soil amendments has been evidenced as a practical strategy to remediate pollution of ARGs in soils. However, little is known about PA effects on horizontal gene transfer (HGT) of ARGs by conjugation. This study investigated the effects of a woody waste-derived PA prepared at 450°C and its three distillation components (F1, F2, and F3) at different temperatures (98, 130, and 220°C) on conjugative transfer of plasmid RP4 within *Escherichia coli*. PA at relatively high amount (40–100 μL) in a 30-mL mating system inhibited conjugation by 74–85%, following an order of PA > F3 ≈ F2 ≈ F1, proving the hypothesis that PA amendments may mitigate soil ARG pollution by inhibiting HGT. The bacteriostasis caused by antibacterial components of PA, including acids, phenols, and alcohols, as well as its acidity (pH 2.81) contributed to the inhibited conjugation. However, a relatively low amount (10–20 μL) of PA in the same mating system enhanced ARG transfer by 26–47%, following an order of PA > F3 ≈ F2 > F1. The opposite effect at low amount is mainly attributed to the increased intracellular reactive oxygen species production, enhanced cell membrane permeability, increased extracellular polymeric substance contents, and reduced cell surface charge. Our findings highlight the hormesis (low-amount promotion and high-amount inhibition) of PA amendments on ARG conjugation and provide evidence for selecting an appropriate amount of PA amendment to control the dissemination of soil ARGs. Moreover, the promoted conjugation also triggers questions regarding the potential risks of soil amendments (e.g., PA) in the spread of ARGs via HGT.

## 1 Introduction

Antimicrobial resistance (AMR) in bacterial pathogens, largely caused by excessive use of antibiotics, has become one of the greatest challenges to human health and the most significant emerging environmental issues in the 21st century. AMR would cause an annual death of approximately 10 million by 2050 and over US$100 trillion economic burden over the next few decades without effective measure.^1^ Increasing studies have shown that ecological niches rich in nutrients and bacterial communities, such as wastewater treatment plants,^2^ rivers,^3^ oceans,^4^ and soils,^5,6^ are ideal settings for the occurrence, evolution, and spread of antibiotic resistance genes (ARGs).^5^ Soil, the most biodiverse habitat containing the most diverse microorganisms in the planetary health system,^6,7^ has become one of the major reservoirs of environmental ARGs with high diversity and abundance.^5,6^ This was mainly ascribed to intensive agricultural practices, including reclaimed water irrigation and applications of manure, sewage sludge, and composts.^7−9^ Furthermore, pollutants (e.g., pesticides, antibiotics, and heavy metals) may also facilitate the evolution and dissemination of ARGs between soil microbiota via selecting the existing genes,^10,11^ stimulating genetic mutation,^12^ and/or promoting horizontal gene transfer (HGT).^13,14^ ARGs enriched in soils can potentially disseminate AMR from soil microbiota to plant microbiota,^15^ thus spreading to humans via food chains.^6,16^ This would further aggravate AMR threats to human health and ecological security.^15^ Hence, it is of increasing significance to alleviate ARG burdens in soil ecosystems and their influences on global health.

Increasing strategies are recommended to alleviate ARG pollution in agricultural soils, such as aerobic composting or anaerobic digestion of manure and sludge before use,^7,17^ inactivation of ARGs and antibiotic resistant bacteria from the reclaimed water before irrigation,^18^ vermiculture,^19,20^ phage therapy,^21^ and soil amendment (e.g., biochar, calcined eggshell, and nanomaterials) applications.^10,22,23^ Among them, soil amendments,^22,23^ which may fight against ARGs already existing in soils, have attracted increasing attention because of their efficiencies and convenient implementation.^23^ Pyroligneous acids (PAs) derived from biomass pyrolysis, as multifunctional soil amendments with long history, can decrease soil salinity and pH,^24,25^ regulate bacterial community,^24^ kill plant pathogenic fungi and bacteria,^26−28^ and promote crop growth and grain yield.^25^ Our recent studies first demonstrated that PA amendment can reduce ARG levels in soils grown with vegetable *Brassica*, regardless individual application or combined with biochar amendment.^10,29^ Similarly, another research also reported that the combined treatment of bamboo biochar and distilled PA showed significant synergistic effects on alleviating ARG proliferation in municipal solid waste composts.^30^ These studies mainly ascribed the decreased ARG abundances to the inhibited HGT, reduced co-selection of heavy metals and lowered potential host bacteria of ARGs but ignored the underlying mechanisms of PA on HGT process. Previous studies have verified that PA as a bacteriostatic agent can damage the cell structure and inhibit the bacterial energy production and cell metabolic activity,^26−28^ which may impede the conjugative process. However, these potential effects and the relevant mechanisms underlying HGT process by PA amendment remain unclear, limiting the further development of the PA-based technology to abating soil ARG pollution.

HGT, including conjugation, transformation, transduction, gene-transfer agents, and cell fusion in prokaryotes,^31,32^ is commonly known to have a great impact on transferring ARGs among bacterial species.^13,33^ Particularly, conjugation, as a major pathway of HGT,^34^ can disseminate ARGs carried on mobile genetic elements such as plasmids, insertion sequences, and transposons^32^ via conjugation bridges formed by a conjugative/sex pilus or membrane pores within intrageneric or intergeneric bacteria.^13,32^ Increasing evidence documented that antibiotics (e.g., cefotaxime, ampicillin, and ciprofloxacin)^35^ and non-antibiotic chemicals (e.g., preservatives, disinfectants, and sweeteners)^36,37^ at sub-inhibitory levels, that is, below the minimum inhibition concentration (MIC), can promote ARG conjugative transfer. The potential mechanisms include the overproduced reactive oxygen species (ROS), enhanced membrane permeability, promoted cell-to-cell contact by stimulating extracellular polymeric substance (EPS) production and decreasing electrostatic repulsion between cell surfaces, induced SOS response, and regulated conjugation-related gene expression.^36−38^ On the contrary, these antibiotics^39^ and non-antibiotic chemicals^36,40^ at inhibitory levels (above the MIC) generally inhibited ARG conjugation by strong bacteriostasis. As common soil amendments, PAs containing a large variety of water-soluble compounds (e.g., 39–124 species) such as organic acids, phenolics, esters, aldehydes, and alcohols^29,41^ can effectively inactivate bacteria,^26^ fungi,^28^ plant pathogens,^27^ and viruses.^42^ A study reported that a PA obtained by slow pyrolysis of a mixture of pine, spruce, and fir wood particles, containing organic acids such as acetic acid and aldehydes like vanillin and furfural, all of which are known as microbial inhibitors, showed antibacterial activity against five pathogenic bacterial strains.^43^ Also, a recent study reported that phenolic compounds (e.g., *p*-nitrophenol, *p*-aminophenol, and phenol), one type of the representative components of PA, at 10–100 mg/L significantly promoted conjugative transfer of plasmid RP4 from *Escherichia coli (E. coil)* HB101 to the bacterial community in activated sludge by increasing ROS levels and bacterial membrane permeability.^44^ Therefore, we hypothesized that PA amendment could induce hormesis on intrageneric conjugative transfer of ARGs, that is, inhibition at high amount by bacteriostasis via antimicrobial components and acidity of PA but promotion at low amount by enhancing ROS production, membrane permeability, and intercellular contact.

To test this hypothesis, the present study investigated the effects of a pristine PA prepared by charring woody waste at 450°C and its three distillation components at different temperatures (98, 130, and 220°C) on the conjugative ARG transfer within the genus *E. coli*. The specific objectives were to (1) investigate the amount effect of PA on the intrageneric conjugation of ARGs, (2) evaluate the roles of its distilled fractions and representative components as well as PA acidity on the conjugation, and (3) explore the potential mechanisms behind the conjugation regulated by PA. This study will expand the insights into PA application as a feasible strategy for controlling the spread of soil ARGs.

## 2 Materials and Methods

### 2.1 Bacterial Strains

Two *E. coli* strains (HB1011 and NK5449) were used to build an intrageneric conjugation model of ARGs mediated by transferable plasmid.^29^*E. coli* strains, belonging to the phylum *Proteobacteria*, were chosen because they are one type of the most abundant bacterial species in soils and other environments.^45^ They are also frequently used as the soil bacterial surrogates.^5,6,38^*E. coli* HB101 harboring transferable plasmid RP4, a typical plasmid named IncP α-type, carries three ARGs against ampicillin (Amp^R^, 100 mg/L), tetracycline (Tet^R^, 16 mg/L), and kanamycin (Km^R^, 50 mg/L). *E. coil* NK5449 carries both the resistance genes for rifampicin (Rif^R^, 160 mg/L) and nalidixic acid (Nal^R^, 50 mg/L).

### 2.2 Preparation and Characterization of PA Samples

PA was pyrolyzed from the blended woody wastes as our previous studies.^10,29^ Three distilled fractions of the pristine PA were obtained by a conventional atmospheric distillation method involving passive volatilization of compounds at 98 (F1), 130 (F2), and 220 (F3)°C as previously reported;^29^ these fractions are commonly used as antimicrobial agents and for soil amendment.^10,29^ It takes more than 6 months to stabilize and relatively purify PA before use as in a previous study,^29^ which would exclude the effect of degradation products of PA and its fractions on conjugation. The properties of pH, density, and chemical composition of the pristine PA and its fractions were characterized previously,^29^ and they are presented in the Supporting Information (Figure S1). Additionally, acetic acid (>99.5% pure), guaiacol (99% pure), 2,6-dimethoxyphenol (98% pure), and 3-methyl-1,2-cyclopentadione (98% pure), four representative components of the PA and the distilled fractions (Figure S1c), were obtained from Sinopharm Chemical Reagent Co., Ltd., China, and Guangzhou Chemical Reagent Factory, China.

### 2.3 Evaluation of Conjugative ARG Transfer in the Presence of PA and Its Fractions

Horizontal ARG transfer mediated by plasmid RP4 between bacteria strains in the presence of PA and its distilled fractions was conducted using an optimized intrageneric conjugative transfer model.^29^ The model was established using *E. coli* HB101 harboring transferable plasmid RP4 as the donor and *E. coli* NK5449 as the recipient in the intrageneric conjugation model. Briefly, after being incubated in 100 mL LB broth medium at 37°C for 12 h with an oscillating speed of 200 rpm, the donor *E. coli* HB101 and recipient *E. coli* NK5449 strains were centrifuged at 4000 rpm and 25°C for 10 min to remove the supernatants. The bacteria pellets were washed twice using phosphate buffer saline (pH 7.2) and then resuspended in LB liquid medium to obtain the desired bacterial concentration (3 × 10^8^ CFU/mL). Subsequently, 150 μL of donor and recipient bacteria suspensions was added into 30 mL LB liquid medium containing different amounts of PA (10, 20, 40, 60, 80, and 100 μL), the three distilled fractions (10, 20, and 40 μL), or four representative components including acetic acid (0.001, 0.01, and 0.1 g/L), 2-methoxy-phenolx (0.01, 0.1, 0.5, and 1 g/L), 2,6-dimethoxy phenol (0.1, 0.5, 1, and 2 g/L), or 3-methyl-1,2-cyclopentanedione (0.05, 0.5, 1.5, and 3 g/L). Notably, the amounts of PA and its three distilled fractions were added according to the recommend agriculture-applied levels (Table S1) and their MICs to the donor and recipient strains (Table S2), and the added amounts of these four components were selected based on the corresponding contents of these four components in PA and its distilled fractions (Figure S1) and their MICs on the donor and recipient strains (Table S2). The nutrition-rich liquid LB system at 37°C may ensure the growth of bacterial donors, recipients, and transconjugants without any nutrient limitation, which could reflect the HGT process in the agricultural soils with a high level of nutrient due to extensive application of chemical fertilizers and manures.^29,46^ After being incubated at 37 °C and 200 rpm for 18 h, the mixtures were appropriately diluted in NaCl (0.9%) and plated on LB-agar plates containing the corresponding antibiotics to select the transconjugants (Amp^R^, Km^R^, Tet^R^, and Rif^R^). After the plates were cultivated at 37°C for 24 h, the transconjugants, donors, and recipients were counted. Then, the conjugative transfer frequency was calculated as the ratio of the transconjugant number to the total recipient number in the control.^14^ Each treatment was set with biological triplicates in three 50 mL sterile conical bottles. The LB-agar plate counting method was used to measure the number of conjugants and conjugative transfer frequency, and three replicate plates were set for each sterile conical bottle to ensure the reproducibility. Each treatment was conducted in nine biological replicates and two repeated operations at least along with the blank control without PA or the fractions.

In parallel, so as to eliminate the interference of bacterial spontaneous mutation and mutation induced by PA and its fractions, both the donor and recipient bacteria exposed to PA and its fractions (0, 10, and 40 μL) were plated onto the transconjugant-selective plates.^46^ Additionally, to confirm the successful transfer of plasmids in donors to recipients, gel electrophoresis was applied to verify the presence of plasmids in transconjugants. The detailed procedures are provided in the Supporting Information (Text S1).

In addition, to verify the effect of PA and its three distilled components on the growth of donor, recipient, and transconjugant strains, three different assays were employed, that is, a LB-agar plate counting method,^47^ a LIVE/DEAD bacterial viability assay method,^47^ and a growth curve testing method.^48^ The 10%, 50%, and 90% MICs (MIC_10_, MIC_50_, and MIC_90_) were assessed by a broth microdilution method.^47^ The detailed procedures are provided in the Supporting Information (Texts S2 and S3).

### 2.4 Determination of PA Acidity Effect on the Plasmid RP4 Conjugative Transfer

To investigate the effect of PA acidity on ARG conjugation, three mating systems with desired pH were set up, including the following: (1) the unadjusted-pH group: the treatments added with PA or its fractions (20 and 40 μL) without any pH adjustment; (2) the adjusted-pH group: the treatments added with PA or its fractions, and the pHs were adjusted to 7.0 as the control group by 0.1 M NaOH; (3) the no-PA group: the treatments without PA or its fraction addition, and the pHs were adjusted as the same as those containing corresponding PA or its fractions by 0.1 M HCl. Besides pH adjustment, other operation procedures of the conjugation for these three treated groups were the same as those in Section 2.3. The pH adjustment could not change the properties of the main components of PA and its fractions, which may only affect the dissociation degree of some PA components, especially acetic acid.

### 2.5 Measurement of ROS

To assess the effect of oxidative stress induced by PA and its fractions on conjugation, the intracellular ROS level was measured by a 2′,7′-dichlorofluorescein diacetate cellular ROS detection assay kit (Beyotime, China).^44^ In addition, 300 μL of glutathione (GSH, a final concentration of 300 μM), a scavenger of ROS, was added into the mating system to examine the role of intracellular ROS overproduction induced by PA in the changed conjugation.^44^ The optimized concentration of GSH (300 μM), which was supersaturated to reduce ROS production and cause little change in the donor and recipient number, was selected according to a preliminary experiment (Figure S2). All experiments were measured with biological triplicates. The detailed procedures are provided in the Supporting Information (Text S4).

### 2.6 Measurement of Cell Morphology, Membrane Permeability, EPS, and Zeta Potential of Bacterial Cells

The bacterial cell morphology in the presence of PA and its distilled components was characterized by transmission electron microscopy (TEM).^36^ Effects of PA and its fractions on cell membrane permeability were further investigated using propidium iodide (Life Technologies, USA) dye.^44^ In addition, 300 μL of GSH (30 mM) was added in 30 mL of 10, 20, or 40 μL PA-exposed cell suspensions to verify the ROS effect induced by PA on the cell membrane permeability. All samples were conducted in biological triplicates. The detailed procedures are provided in the Supporting Information (Text S5).

The heat extraction method was used to obtain EPS of bacteria as described by previous research.^38^ The contents of protein and polysaccharide in the extracted EPS solution, two representative dominant components of EPS and playing important roles in cell surface hydrophobicity and bacterial adhesion, were, respectively, analyzed using a Detergent Compatible Bradford Protein Assay Kit (Beyotime, China) and phenol-sulfuric acid method.^49^ The details are described in the Supporting Information (Text S6).

The zeta potentials of *E. coli* cells exposed with PA and the fractions were measured using a ZS90 zetasizer (Malvern, UK).^14^ Briefly, the bacterial suspension treated with 20 and 40 μL of PA or its distilled components in the mating system was washed with sterilized Milli-Q water twice and the concentration was adjusted to 10^7^ CFU/mL using sterilized Milli-Q water. Finally, the zeta potentials of bacterial suspension were measured.

### 2.7 Statistical Analysis

All experimental data were expressed as mean ± standard deviation (*n* ≥ 3). One-way analysis of variance (ANOVA) with Duncan’s multiple-comparison test (*P* < 0.05) and independent-samples *t*-test (*P* < 0.05) was adopted for significance analysis by the Statistical Product and Service Solutions software (SPSS 20.0, SPSS Inc., Chicago, IL, USA). Pearson correlation analysis was performed by R software (version 4.0.3) with the “corrplot” package. Probit model was used to calculate the MIC_10_, MIC_50_, and MIC_90_ by SPSS 20.0.^47^ The bacterial growth curves were fitted using logistic growth model with Origin 2022.^48^

## 3 Results and Discussion

### 3.1 Hormesis of PA on the Conjugative Transfer of Plasmid RP4

**Figure 1:**
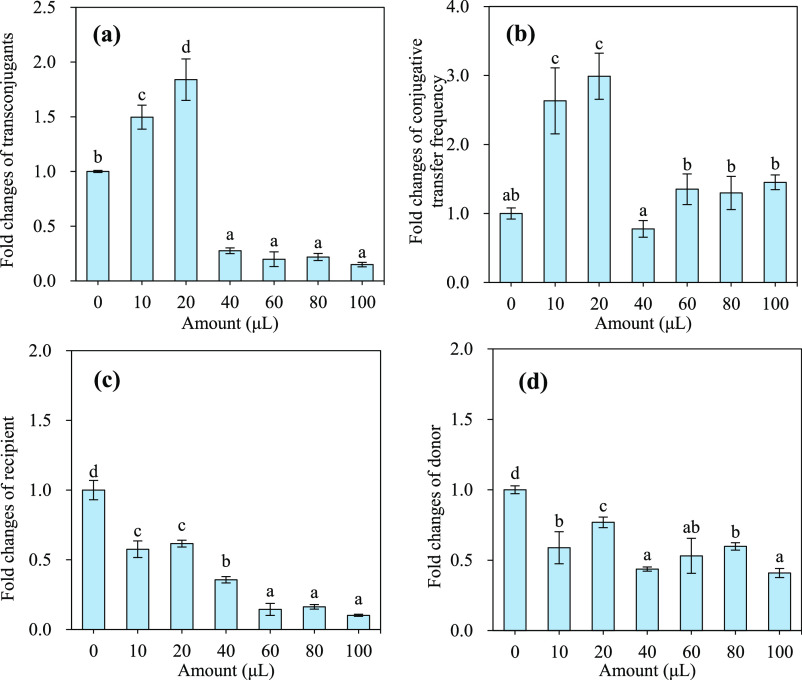
Amount effect of pristine PA on the conjugative transfer of plasmid RP4 between the donor *E. coli* HB101 and recipient *E. coli* NK5449 in a 30-mL mating system. (a) Transconjugant count, (b) conjugative transfer frequency, (c) recipient *E. coli* NK5449 count, and (d) donor *E. coli* HB101 count. PA: the pyroligneous acid prepared from pyrolysis of blended woody waste collected from furniture factories at 450°C for 6 h. The mating conditions of conjugation: 10^8^ CFU/mL *E. coli* HB101 as the donor and 10^8^ CFU/mL *E. coli* NK5449 as the recipient, which were mixed at a volume ratio of 1:1 and incubated at 37 °C for 18 h. The different small letters reflect a significant difference among the different treatments (Duncan’s multiple-comparison test, *n* = 3, *P* < 0.05).

To test the effects of PA and its fractions on ARG conjugation, an LB-agar plate-based conjugation mating system was conducted. The results showed that PA at a relatively high amount of 40–100 μL (above the MIC_10_ levels of 0.706 and 0.807 μL/mL, that is, 21.2 and 24.2 μL/30 mL) in the mating system significantly decreased the transconjugant number by 74–85% (from 3.53 × 10^5^ CFU/mL to 5.24 × 10^4^–9.14 × 10^4^ CFU/mL) compared with the control (Figure 1a and Table S3). Inconsistently, PA significantly promoted the conjugative transfer frequency by 30–45% at 60–100 μL relative to the control (Figure 1b). This plausible enhancement was mainly ascribed to the relatively greater reduction in recipient bacteria number (by 86–90%) than the transconjugant number (by 78–85%) in the presence of PA (Figure 1a,c). As a mixture containing many antimicrobial components, PA has an MIC_50_ and MIC_90_ of 2.78 and 4.85 μL/mL for *E. coli* NK5449 and 2.40 and 4.00 μL/mL for *E. coli* HB101, respectively (Table S2). Thus, at a relatively high concentration of 60–100 μL, ranging between MIC_50_ and MIC_90_ (Table S1), the decreased abundance and activity of recipient and donor bacteria may substantially decrease the transconjugant number due to the antimicrobial effects (Figure 1c,d). This also can be supported by the reported studies, showing that silver ions, silver nanoparticles, and triclosan resulted in the reduction of horizontal transfer frequency above the MIC levels.^39^ However, PA at relatively low amounts of 10 and 20 μL (below the MIC_10_ levels of 0.706 and 0.807 μL/mL, that is, 21.2 and 24.2 μL/30 mL) significantly increased the transconjugant number by 26–47% (from 3.53 × 10^5^ CFU/mL to 4.43 × 10^4^–5.19 × 10^4^ CFU/mL) and the conjugative transfer frequency by 114–234% (from 6.79 × 10^–5^ to 1.45 × 10^–4^–2.27 × 10^–4^) (Figure 1a,b and Table S3). Furthermore, the successful transfer of donor plasmids to recipients was verified by gel electrophoresis, showing clear bands of plasmids in the transconjugants with the same size as those in the donor bacterial strains (Figure S3a). In addition, no mutated bacteria was observed on the transconjugant selection plates (Figure S3b–d), further confirming that the resistance acquisition of the *E. coli* colonies on the selective transconjugant plates was caused by the generation of transconjugants, rather than the spontaneous mutation of bacterial strains exposed with PA. Taken together, these results proved our hypothesis that PA amendment at the selected amount had a hermetic response (low amount, promotion; high amount, inhibition) on intrageneric conjugative transfer of ARGs.

Notably, the PA-promoted conjugative transfer also triggers the questions regarding the potential risks of amendments (e.g., PA, biochar) playing non-negligible roles in spread of ARGs via HGT in soils or environments with similar characteristics (e.g., composts, sediments), particularly under the inappropriate amount. However, a study proposed that low concentrations (1–5 mg/L) were the appropriate amounts for CeO_2_ nanoparticles, one of the typical nanotechnology-enabled products in the agriculture industry applied to enhance crop yield, food safety, and nutritional value,^50^ to control horizontal ARG transfer in soils.^38^ Whereas, the opposite effect was observed at high concentrations (25 and 50 mg/L), even though the donor and recipient bacterial concentrations were decreased.^38^ These distinct amount-dependent patterns of ARG conjugative transfer are mainly attributed to the different chemical characteristics (mixed chemicals vs nanoparticles) of these soil amendments, which could regulate ARG conjugation by influencing bacterial growth and population.^38,39^ Recently, a study evidenced that different types of dissolved biochars, the water-soluble components of biochars, at different amounts (1–100 mg/L) posed significantly inhibited or promoted effects on conjugation of plasmid RP4.^40^ The effects were consistent with that of PA, which is mainly explained by the complex compositions of dissolved biochars such as humic acid-like substances, small molecule phenols, and organic acids. PAs, one of the by-products simultaneously derived from biomass pyrolysis for biochar production,^28,29^ containing similar chemical components with the dissolved biochars (Figure S1), would be responsible for the hormesis on ARG transfer, which will be discussed in the following section.

### 3.2 Representative Components of PA Contributed to the Changed Conjugation

**Figure 2:**
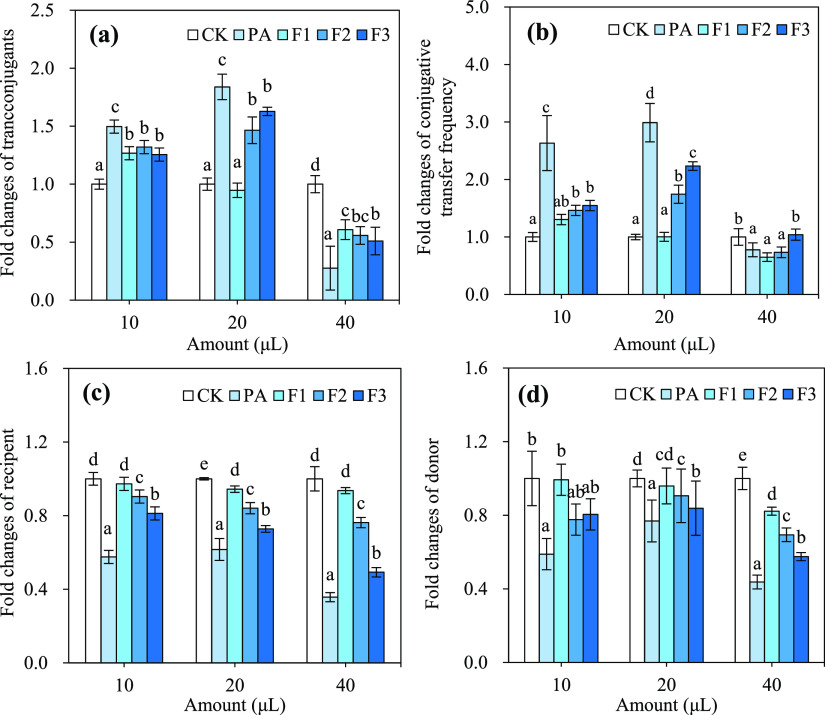
Effects of PA and its distilled fractions on the conjugative transfer of plasmid RP4 between the donor *E. coli* HB101 and recipient *E. coli* NK5449 in a 30-mL mating system. (a) Transconjugant count, (b) conjugative transfer frequency, (c) recipient *E. coli* NK5449 count, and (d) donor *E. coli* HB101 count. CK: the mating system of conjugation without any PA or its distilled fractions; PA: the pyroligneous acid prepared from pyrolysis of blended woody waste collected from furniture factory at 450°C for 6 h; F1, F2, and F3: the fraction of PA collected using atmospheric distillation at 98, 130, and 220°C, respectively. The mating conditions of conjugation: 10^8^ CFU/mL *E. coli* HB101 as the donor and 10^8^ CFU/mL *E. coli* NK5449 as the recipient, which were mixed at a volume ratio of 1:1 and incubated at 37°C for 18 h. The different small letters represent a significant difference among the different treatments at the same amount (Duncan’s multiple-comparison test, *n* = 3, *P* < 0.05).

To distinguish the chemical components of pristine PA playing important roles in the conjugation, three distilled fractions of PA were first obtained at different temperatures of 98, 130, and 220°C to examine their effects on the conjugative transfer. Similar to the pristine PA, these three distilled fractions also showed hormesis effects on the conjugative transfer (Figure 2). For example, F3 decreased the transconjugant number by 49% (from 3.43 × 10^5^ CFU/mL to 1.74 × 10^5^ CFU/mL) at a relatively high amount (40 μL) but increased the transconjugant number by 25–63% (from 4.64 × 10^5^ CFU/mL to 5.82 × 10^5^ CFU/mL in 10 μL and from 3.44 × 10^5^ CFU/mL to 5.59 × 10^5^ CFU/mL in 20 μL) at relatively low amounts (10 and 20 μL) (Figure 2a,b and Table S4). Moreover, the hormesis effects of PA and its fractions on the conjugation showed that PA had a stronger degree of inhibition or promotion than its three fractions (Figure 2a,b). These results were in line with our previous research,^29^ that is, the exposure of PA, F2, and F3 at selecting appropriate amounts effectively decreased ARG abundances in the rhizosphere and bulk soils, while F1 showed an opposite effect.^29^ These results also further confirmed the feasibility of PA amendments for mitigated soil ARG dissemination via inhibiting HGT process. As previously characterized, the compositions of PA and its distilled fractions contained varied chemical compounds (e.g., acids, phenols, and alcohols) with different relative contents (Figure S1b,c). Thus, the promoted effect at a relatively low amount on the transfer showing the order of PA > F3 ≈ F2 > F1 and the inhibited effect at a relatively high amount on the transfer showing the order of PA > F3 ≈ F2 ≈ F1 indicated the different roles of PA components in the conjugative transfer.

**Figure 3:**
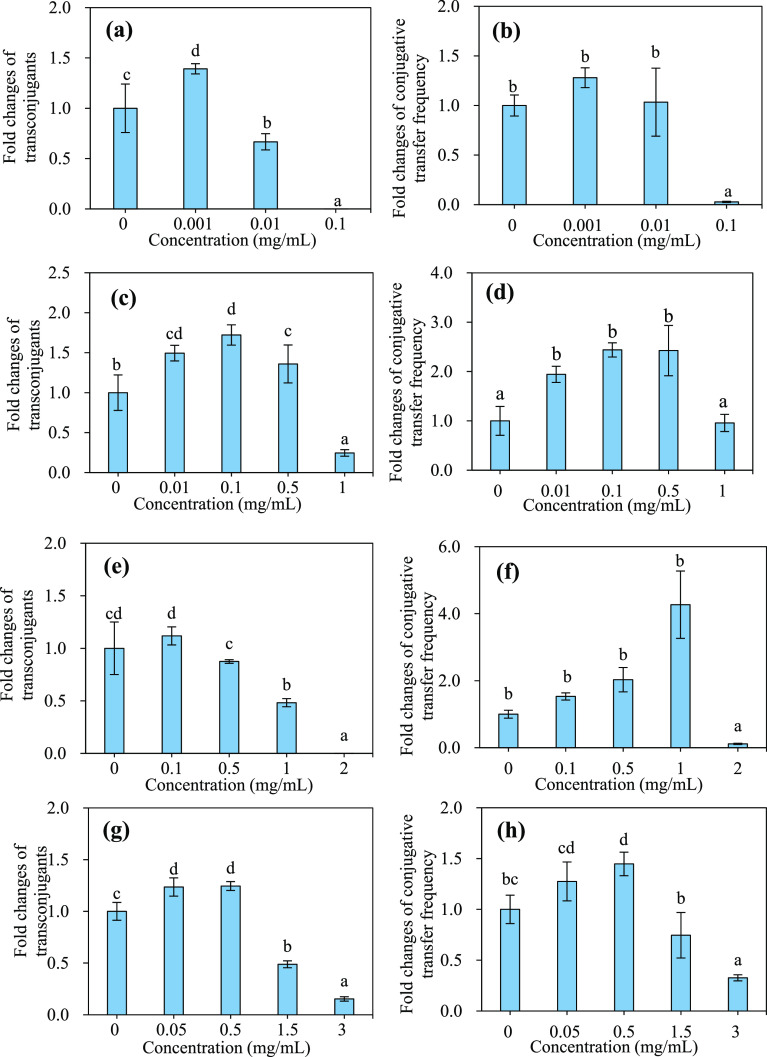
Effects of the representative chemical components of PA on the conjugative transfer of plasmid RP4 between the donor *E. coli* HB101 and recipient *E. coli* NK5449 in a 30-mL mating system. (a,b) Acetic acid, (c,d) 2-methoxy-phenolx, (e,f) 2,6-dimethoxy-phenol, and (g,h) 3-methyl-1,2-cyclopentanedione. The mating conditions: 10^8^ CFUcfu/mL *E. coli* HB101 as the donor and 10^8^ CFU/mL *E. coli* NK5449 as the recipient, which were mixed at a volume ratio of 1:1 and incubated at 37°C for 18 h. The different small letters represent a significant difference among the treatments exposed with the individual component at the same concentration (Duncan’s multiple-comparison test, *n* = 3, *P* < 0.05).

To distinguish the roles of individual PA components in the conjugative transfer, four representative components of PA, including acetic acid, two phenolic derivatives (2-methoxy-phenolx and 2,6-dimethoxy phenol), and 3-methyl-1,2-cyclopentanedione, determining PA functionality as a antimicrobial agent, antioxygen, a plant growth regulator, and a feed supplement,^24,25^ were selected according to their relative contents (Figure S1). These four representative components also showed the hormesis effects on the transfer of plasmid RP4 (Figure 3), similar to PA and its distilled fractions (Figure 2). The four components at sub-MIC_10_ concentrations (0.001 mg/mL for acetic acid, 0.01 mg/mL for 2-methoxy-phenolx, 0.1 mg/mL for 2,6-dimethoxy phenol, and 0.05 mg/mL for 3-methyl-1,2-cyclopentanedione) promoted the transconjugant number, respectively, by 39%, 49%, 12%, and 24%, following an order of acetic acid ≈ 2-methoxy-phenolx > 2,6-dimethoxy phenol ≈ 3-methyl-1,2-cyclopentanedione (Figure 3 and Table S5). Contrarily, when the amounts of these four components increased to the inhibitory levels (i.e., 0.1 mg/mL for acetic acid, 1 mg/mL for 2-methoxy-phenolx, 2 mg/mL for 2,6-dimethoxy phenol, and 3 mg/mL for 3-methyl-1,2-cyclopentanedione), the transconjugant number significantly decreased and followed an order of acetic acid ≈ 2,6-dimethoxy phenol > 3-methyl-1,2-cyclopentanedione > 2-methoxy-phenolx (Figure 3, Tables S2 and S5). In previous studies, these hormesis effects were also observed for antibiotics (e.g., gentamicin, sulfamethoxazole, and tetracycline)^39^ and phenolic compounds (e.g., chloroxylenol and *p*-nitrophenol).^44,51^ However, free nitrous acid at the subinhibitory concentrations (<0.02 mg N/L) showed inhibited effects on plasmid RP4 conjugation within genera of *E. coli* K12 and *E. coli* HB101, caused by the limited adenosine triphosphate production and the down-regulated transfer genes via releasing intracellular Fe^2+^ and decreasing intracellular Mg^2+^ levels.^52^ These results confirmed that the selected four representative components of PA variedly contributed to the hormesis effects on the conjugation.

Besides the concentrations or amount-dependent effects, the effects of these chemicals on conjugation are mainly shaped by their structure-dependent antibacterial activity,^46,47^ determining bacterial activity, oxidative stress, cell membrane permeability, and cellular metabolism.^38,47,52^ The antibacterial activity of PA was stronger than that of its fractions (Figures 2c,d, S4, S5, Table S2, S5, Text S7), consistent with the orders of hormesis effects induced by them on the conjugative transfer (Figure 2). This suggested that the antibacterial activity of PA and its distilled components to the donor and recipient bacteria played critical roles in inhibiting or promoting intrageneric conjugation. Numerous research studies showed that the antibacterial properties of organic acids (e.g., acetic acid, oleanolic acid, and ursolic acid), phenols (e.g., 2-methoxy-phenolx, eugenol, and carvacrol), and ketones (e.g., formoxanthone, macluraxanthone, and xanthone) in PA may kill pathogens (e.g., *E. coli*, *Staphylococcus aureus*, and *Bacillus subtilis*) by damaging the cell membrane, inhibiting energy metabolism, and denaturizing protein.^26,53^ In the present study, the four representative components effectively reduced the number of donor and recipient bacteria at the inhibitory levels, showing the antibacterial activity order of acetic acid > 2,6-dimethoxy phenol > 3-methyl-1,2-cyclopentanedione > 2-methoxy-phenolx (Figure S6). Additionally, the statistically significant correlations were observed between the contents of these four representative components and the inactivation of donor or recipient strains (Figure S7), confirming that the bacteriostasis of these PA components at high amounts resulted in the inhibition of conjugation transfer. However, the contents of these four representative components showing an order of F3 (85.6%) > F2 (68.2%) > F1 (67.4%) > PA (58.7%) (Figure S1c) are not fully consistent with the orders of the inhibited conjugation. These results indicated that the potential synergistic effect of the individual components on the antimicrobial activity of PA partly played roles in regulating the conjugation, which should be examined in future. Consistently, the sub-MIC_90_ levels of acetic acid (0.001 mg/mL), 2-methoxy-phenolx (0.01–0.5 mg/mL), 2,6-dimethoxy phenol (0.1 mg/mL), and 3-methyl-1,2-cyclopentanedione (0.05–0.5 mg/mL) posed promotive effects on the conjugation (Figure 3). This was also supported by previous studies,^44,51^ which reported that phenolic compounds such as *p*-nitrophenol, *p*-aminophenol, phenol, and chloroxylenol at sub-inhibitory levels of 10–100 mg/L promoted the plasmid RP4 transfer from *E. coli* HB101 to the bacteria in activated sludge. Collectively, these results evidenced that these four representative components of PA showing responses of non-lethal stress at sub-MIC_90_ levels and bactericidal effects at inhibitory levels contributed to the PA-induced hormesis effect on ARG conjugation. However, the evaluation of absolute concentrations of the representative components in PA and its fractions may bring more reference significance for selecting an appropriate dosage of PA amendment in the ARG-polluted soils, which should be further studied. Additionally, stabilized and relatively purified PA was prepared in this study, which would exclude the effect of degradation products of PA and its fractions on conjugation. Notably, besides these four components, other individual components (e.g., benzene, aldehydes, and esters) in PA (Figure S1b,c) or degradation products (e.g., 3,3′,5,5′-tetramethoxy-1,1′biphenyl-4,4′-diol) of fresh PA also exhibited antibacterial activity, probably contributing to the hormesis effects on the conjugation; this cannot be excluded in the present study, which needs to be further examined.^26,28,53^

### 3.3 Reduction of pH on Decreased the Conjugative Transfer of ARGs

**Figure 4:**
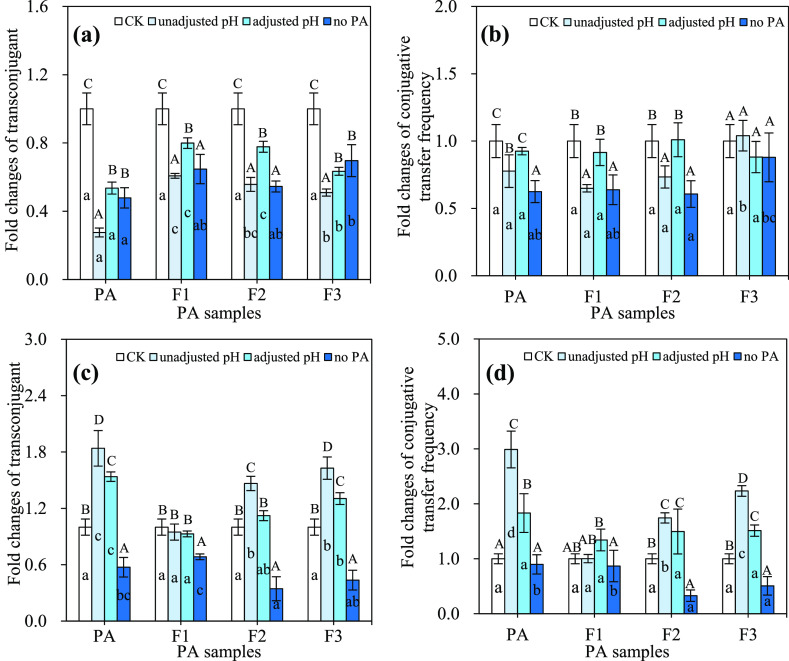
pH effects of PA and its distilled fractions on the conjugative transfer of plasmid RP4 between *E. coli* HB101 and NK5449. (a) Transconjugant count and (b) conjugative transfer frequency at the amount of 40 μL in a 30- mL mating system, (c) transconjugant count, and (d) conjugative transfer frequency at the amount of 20 μL in a 30-mL mating system. Unadjusted pH: the treatments added with PA or its fractions without any pH adjustment; adjusted pH: the treatments added with PA or its fractions, of which pHs were adjusted to 7.0 as the control group (CK); no PA: treatments without PA or its fraction addition, of which pHs were adjusted as the same as those containing the corresponding PA or its fractions. PA: the pyroligneous acid prepared from pyrolysis of blended woody waste collected from a furniture factory at 450°C for 6 h; F1, F2, and F3: the fraction of PA collected using atmospheric distillation at 98, 130, and 220°C, respectively. The mating conditions: 10^8^ CFU/mL *E. coli* HB101 as the donor and 10^8^ CFU/mL *E. coli* NK5449 as the recipient, which were mixed at a volume ratio of 1:1 and incubated at 37°C for 18 h. The different small letters represent a significant difference among the different treatments of PA and its fractions, and the capital letters indicate a significant difference among the treatments with and without pH adjustment (Duncan’s multiple-comparison test, *n* = 3, *P* < 0.05).

The process of ARG conjugative transfer is highly sensitive to external environmental conditions (e.g., pH, temperature, humidity, salinity, and oxygen).^54−56^ The acidic components of PA, such as acetic acid and 2-methoxy-phenol (Figure S1b,c), endow PA and the distilled fractions with the acidic characteristics (pH ≤ 2.81) (Figure S1a), which significantly lowered the pH values (5.45–6.75) of the mating systems relative to the control (pH 7.0) (Table S6). Thus, it is reasonable to assume that the acidic pH of PA could play an important role in inhibiting conjugation. To test this hypothesis, three conjugation experiments with desired pHs were conducted (Figure 4). At a relatively high amount (40 μL) of PA and its fractions, the ARG conjugation was significantly promoted in the adjusted-pH group (pH 7.0) compared to the unadjusted-pH group (both groups treated with same PA but different pH) (Figure 4a,b). Moreover, the transconjugant number and conjugative transfer frequency decreased by 30–52 and 12–39%, respectively, in the no-PA group (pH 5.45–6.45) compared to CK (Figure 4a,b). These results confirmed that the acidic PA-lowered pH of mating systems under the high amount inhibited the conjugation of plasmid RP4. At a relatively low amount (20 μL) of PA and its fractions, the conjugative transfer was still significantly inhibited in the adjusted-pH group (pH 7.0) relative to the unadjusted-pH group (Figure 4c,d). In addition, the transconjugant number and conjugative transfer frequency in the no-PA group (pH 6.07–6.75) decreased by 32–66 and 10–67% compared to CK (pH 7.0) and significantly decreased by 26–127 and 13–209% compared to the unadjusted-pH group, respectively (Figure 4c,d). These results further suggested that the PA-lowered mating pH under the low amount also partially inhibited the ARG conjugation. The pH-inhibited effect was further corroborated by the significant positive correlations between pHs of the mating systems and fold changes of transconjugant number (Figure S7). Collectively, these results proved the hypothesis that the decreased mating pH by acidic PA played a crucial role in inhibiting the conjugation of ARGs between *E. coli*, regardless of the high or low amount of PA.

Particularly, pH is generally closely related to bacterial abundance and activity,^57,58^ the key factors regulating ARG conjugation.^7,17,57^ Under the high amount of PA and its fraction except for F1, the recipient and donor numbers significantly decreased by 24–64 and 31–56%, while their number partially restored after adjusting the mating pH at 7.0, and decreased by 9–21 and 4–24% in the no-PA group compared to CK, respectively (Figure S8). Moreover, the statistically significant correlations were observed between the mating pH and fold changes of recipient and donor numbers (Figure S7). These results evidenced that the decrease in matting pH by a high amount of PA caused strong bacteriostasis. Extensive studies revealed that the *E. coli* strains in acidic conditions (pH 4.0 and 5.5) generally resulted in low bacterial abundance and activity via destroying the tertiary structure of the membrane protein by electrostatic effects^59^ and suppressing DNA replication by DNA depurination.^60^ The PA acidity is determined by its acidic components, such as acetic acid, 2-methoxy-phenol, 2,6-dimethoxy phenol, and 3-methyl-1,2-cyclopentanedione, which also had antibacterial activity at inhibitory levels (Figure S6 and Table S7). For example, acetic acid reduced the number of donors and recipients by 99% at 0.1 mg/mL, and 2-methoxy-phenol respectively reduced by 75 and 83% at 1 mg/mL (Figure S6a–d). These results further confirmed that the acidic components of PA such as acetic acid, 2-methoxy-phenol, 2,6-dimethoxy phenol, and 3-methyl-1,2-cyclopentanedione under high levels inhibited conjugation by their strong bacteriostasis. Consistently, a study demonstrated that acidification of manure from neutral (7.4) to acidic pH (4.8–5.4) promoted the degradation of sulfonamide antibiotics and decreased *sul* genes and sulfonamide-resistant bacteria (e.g., *Xanthomonadaceae*, *Pseudomonadaceae*, and *Yaniellaceae*).^61^ Thus, the risk of conjugative *sul* gene transfer was reduced, which was facilitated to reduce ARG risk in soils.^61^ On the contrary, another study found that the potential of gene transfer via genetic vectors (e.g., plasmids, extracellular DNA, and phages) was promoted at acidic pH 4.0 and 5.0 but inhibited at alkaline pH 9.0 and 10.0, mainly attributed to the enhanced or inhibited propagation of tetracycline-resistant bacteria.^57^ These inconsistent effects of acidic pH on the conjugation were mainly attributed to the different pH sensitivities of the tested resistant strains (*Pseudomonadaceae* vs *Acetobacter*) in the two studies. However, in the present study, the *E. coli* strains showed poor adaptability to acidic pH via the decreased bacterial activity in low pH (5.45–6.45) caused by PA, which first provided the direct evidence that the acidic characteristic of PA amendment can inhibited the conjugative transfer of ARGs via its bacteriostasis.

Notably, although the pHs of the adjusted-pH group were the same as the CK groups, the transconjugant number in the adjusted-pH group under a high amount of PA was still lower compared to CK (Figure 4). Furthermore, the pHs of no-PA group were adjusted to the same as the unadjusted-pH group, but the transconjugant number of no-PA group was higher under a high amount of PA or lower under a low amount of PA compared to the unadjusted-pH group (Figure 4). These results implied that the decreased pH of the mating systems resulting from PA acidity is not the only contributor for inhibiting or promoting the conjugation and further verified that the PA components (e.g., acids, phenols, and ketones) also played important roles in the conjugation. Previous studies reported that a variety of organic compounds (e.g., dicamba, *p*-aminophenol, and phenol) at sub-inhibitory levels could promote ARG conjugation by the increased ROS production, membrane permeability, and intercellular contact but inhibited the conjugation via bacteriostasis above the MIC levels.^14,44^ Therefore, the promoted conjugation by PA may be regulated by (1) intracellular ROS production,^51^ (2) membrane permeability,^44^ and (3) intercellular contact,^38^ but the inhibited conjugation by PA may be regulated by (1) the inhibited recipient and donor growth, (2) destroyed antioxidant system, (3) damaged cell membrane, and (4) broken pilus, which are further verified in the following sections.

### 3.4 Increased ROS Production by PA

**Figure 5:**
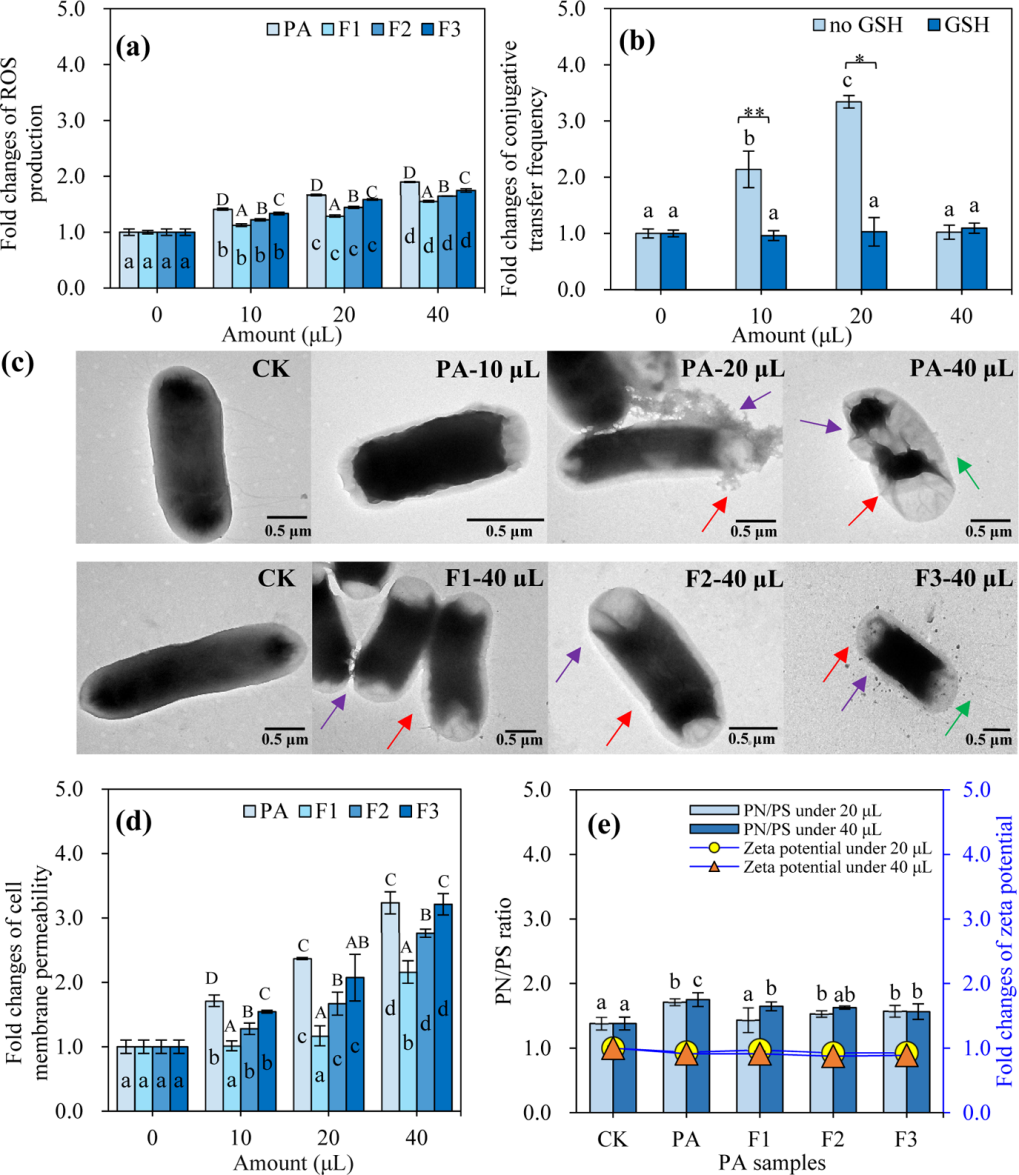
Effects of PA and its distilled fractions on (a) intracellular ROS production of the mixed donor and recipient in a 30-mL mating system. Effect of ROS scavenger GSH on (b) conjugative transfer frequency of plasmid RP4 between *E. coli* HB101 and NK5449 under PA exposure. (c) Representative TEM images of the mixed *E. coli* strains exposed to PA and its fractions. Red arrows: the damages of cytomembrane; green arrows: fractured pilus; purple arrows: shrunken or leaky cytoplasm. Fold changes in (d) cell membrane permeability of the mixed *E. coli* strains exposed PA and its fractions. Effects of PA and its distilled fractions on (e) PN/PS ratio (left vertical axis) and fold changes in zeta potential (right vertical axis) of the mixed *E. coli* strains. The culture conditions: 10^8^ CFU/mL *E. coli* HB101 as the donor and *E. coli* NK5449 as the recipient, which were mixed at a volume ratio of 1:1 and incubated at 37°C for 18 h. The different small letters represent a significant difference among the different treatments added with PA or the fractions at different amounts, the capital letters indicate a significant difference among the treatments added with PA and its fractions with the same amounts (Duncan’s multiple-comparison test, *n* = 3, *P* < 0.05), and the asterisks indicate significant differences between the treatments with and without GSH addition (independent sample *t*-test, *n* = 3, * for *P* < 0.05, ** for *P* < 0.01).

ROS in bacterial cells induced by external environment stress, including ^•^O_2_^–^, ^•^OH, and H_2_O_2_, could stimulate oxidative stress, break biomolecules (e.g., DNA, membrane proteins, and lipids), activate SOS response, and even cause cell death, thus regulating the ARG conjugation.^14,51^ In this study, the amount-dependent increase in intracellular ROS levels was found in the *E. coli* strains exposed with PA and the fractions, and the increasing degree of ROS via PA was stronger than its three fractions (Figure 5a), in line with their promoted or inhibited effects on the conjugation (Figure 2a,b). Importantly, positive correlations were obtained between the fold changes of transconjugants and ROS levels at low amounts (10 and 20 μL) of PA and the fractions (Figure S7). ROS may directly break double-strand DNA or indirectly damage DNA by oxidizing the deoxynucleotide pool, inducing SOS response and repair mechanisms to enhance the successful recombination of exogenous genes,^62^ thus stimulating the conjugation process.^14,63^ These results confirmed that the low amount of PA and the fractions promoted the conjugation via the ROS overproduction. The overproduction of intracellular ROS could induce oxidative stress, thus disrupting the intracellular redox balance.^62^ Notably, although PA and the fractions induced the higher ROS levels at a high amount of 40 μL than the low amounts of 10 and 20 μL (Figure 5a), the conjugative transfer of plasmid RP4 under the high amount was much lower than those of the low amount (Figures 1a and 2a). This was mainly attributed the bacterial cell death stress by the high level of ROS (Figures 2c,d and S4, S5). To further determine whether the observed increase in ROS production was related to the intrageneric conjugative transfer, the effect of GSH, a widely used ROS scavenger,^64^ on plasmid RP4 conjugation was examined (Figure 5b). Expectedly, the GSH addition significantly decreased the conjugative transfer frequency at relatively low amounts (10 and 20 μL) relative to the treatments without GSH but showed a non-significant difference among the CK and PA treatments (Figure 5b), confirming that the increased moderate ROS level of *E. coli* induced by relatively low amounts of PA exposure stimulated the conjugation of plasmid RP4 within the genus *E. coli*. Additionally, for the relatively high amount (40 μL), after adding GSH, the conjugative transfer frequency and the number of recipient and donor cells also had no significant difference with those in the control without PA group (Figures 5b and S9), further confirming that a high amount of PA and its fractions resulted in cell death by exceeding ROS levels to inhibit the conjugation.

To further investigate the potential relationship between ROS overproduction and PA components, Pearson correlation analysis was conducted (Figure S7). The fold changes of ROS levels positively correlated with the contents of acetic acid, 2-methoxy-phenolx, 2,6-dimethoxy phenol, and 3-methyl-1,2-cyclopentanedione of PA (Figure S7), implying that these four representative chemical components play important roles in the ROS overproduction. Extensive studies showed that phenolic compounds such as thymol, eugenol, and carvacrol could destroy the antioxidant defense systems of bacterial cells by decreasing antioxidant enzyme activity (e.g., catalase and superoxide dismutase) and causing excessive ROS accumulation.^53,65^ These results displayed that the representative chemical components, particularly phenolic compounds such as 2-methoxy-phenolx and 2,6-dimethoxy phenol detected in the PA, played important roles in the ROS overproduction. Furthermore, the acidic conditions could also enhance the ROS accumulation by inhibiting antioxidant enzyme activity (e.g., the alkyl hydroperoxide reductase and cytochrome *o* oxidase) and destroying electron transport chains in bacterial cells, resulting in leakage of electrons to oxygen prematurely and ROS formation.^66,67^ Pearson correlation coefficient analysis showed the negative correlation between the fold changes of ROS levels and the pH values (Figure S7), implying that the decreased pH caused by acidic PA also induced the ROS overproduction in *E. coli* cells. Together, these results validated that the moderate ROS level of *E. coli* induced by PA at relatively low amounts promoted the RP4 plasmid-mediated conjugative transfer within *E. coli* genera, while the exceeding level of ROS induced by high amounts of PA resulted in cell death to inhibit the conjugation, and the ROS overproduction was mainly due to the representative components of PA (i.e., acids, phenols, and ketones) and the decreased pH.

### 3.5 Increased Cell Membrane Permeability by PA

Bacterial cell membrane is an important barrier to prevent extracellular chemical materials and genes from free accessing cells.^31^ The cell membrane structure of the mixed donor and recipient strains exposed to PA and its fractions was first assessed by TEM (Figures 5c and S10). In the untreated group, the *E. coli* strains showed a normal cellular structure with intact cell membranes, a smooth surface, and compact cytoplasm, and the cells were dispersed with limited physical contact (Figures 5c and S10). At the low amounts (10 and 20 μL) of PA, the cells were obviously observed with damaged cell membranes, emerged pores, unclear cell boundaries, and enhanced cell-to-cell contact (Figures 5c and S10a), implying that the bacterial membrane permeability enhanced, which may favorable to ARG conjugation.^51^ Furthermore, the bacterial membrane permeability was further quantified using flow cytometry (Figure 5d). Specifically, the amount-dependent enhancement of membrane permeability was found in *E. coli* strains exposed with low amounts (10 and 20 μL) of PA and the fractions, and PA had a stronger degree of enhancement on the membrane permeability than its three fractions (Figure 5d), consistent with the promoted effect on the conjugative transfer (Figure 2a,b). Furthermore, positive correlation was found between the fold changes of cell membrane permeability and transconjugants exposed with PA and its fractions (Figure S7). These results demonstrated that the low amount of PA and fractions promoted the conjugation via the enhanced cell membrane permeability (Figures 1a and 2a). Notably, although PA and the fractions induced the higher levels of cell membrane permeability at a high amount of 40 μL than the low amounts of 10 and 20 μL (Figure 5d), the number of transconjugants, donors, and recipients under the high amount is much lower than those of the low amount (Figure 2a,c,d). This further implied that the bacteria cells were completely inactivated and damaged via the completely ruptured cell membrane by the high amount of PA (Figures 5c and S10). Consistently, this was also why more severely damaged cell membrane and shrunken or leaky cytoplasm were found in the TEM images (Figures 5c and S10), supported by the LCSM images of the donor and recipient (Figure S4) and bacteria growth curves of the donor, recipient, and transconjugant (Figure S5 and Table S8). Moreover, bacterial pilus generally takes charge of competence for DNA uptake, motility, and cell surface adhesions,^68^ which may facilitate the resistance plasmid transfer between bacteria. The complete pilus was found on the surface of untreated bacterial cells in the control group, while obvious pilus fractures were observed for the bacterial cell treated with the high amount of PA (Figures 5c and S10), thus impeding the successful transfer of ARGs. These results demonstrated that a moderate increase in cell membrane permeability induced by a low amount of PA facilitated the conjugative ARG transfer, while the severe cell membrane damage, pilus fracture, cytoplasmic shrinkage, and leakage caused by a high amount of PA resulted in cell death to inhibit conjugation.

ROS could react with polyunsaturated fatty acids located on bacterial cell membrane, leading to excessive oxidation of lipids, thereby enhancing the membrane permeability to facilitate the plasmid transfer from donor to recipient bacteria.^62^ To further verify the increased bacterial membrane permeability induced by ROS overproduction under PA exposure, the membrane permeability of bacterial cells added with GSH was examined. Regardless of the low or high amount of PA exposure, the cell membrane permeability in the GSH treatments was significantly lower relative to those without GSH addition and showed no significant difference compared with those in the control treatment without PA addition (Figure S11). Additionally, significant positive correlations were observed between the fold changes of ROS and cell membrane permeability (Figure S7). These results illustrated that the increased ROS levels induced by PA exposure enhanced the membrane permeability of the *E. coli* strains. Previous studies have shown that sub-inhibitory concentrations (0.04–1.00 mg/L) of chloroxylenol, as an aromatic organic compound containing phenol, could increase cell membrane permeability by the membrane lipid peroxidation to stimulate the plasmid RP4 transfer between *E. coli* DH5α and *Pseudomonas* HLS-6.^51^ Toxic chemicals, particularly hydrophobic molecules (e.g., 2-methoxy-phenolx and 2,6-dimethoxy phenol), could accumulate within the membrane lipids, participate in chemical interactions between the fatty acyl chains, and damage the membrane phospholipids to facilitate cell membrane permeability.^69^ Furthermore, positive correlations were found between the fold changes of cell membrane permeability and the contents of acetic acid, 2-methoxy-phenolx, 2,6-dimethoxy phenol, and 3-methyl-1,2-cyclopentanedione in PA (Figure S7). These results displayed that these four representative components detected in the pristine PA contributed to the increased cell membrane permeability. Additionally, the acidic conditions also could enhance the cell membrane permeability by increasing the proportion of unsaturated fatty acids, particularly C18-1w9c and C19-Cyc, which were the key metabolites in the synthesis of fatty acids and the substrates for the synthesis of unsaturated fatty acids.^70^ The negative correlation was observed between the fold changes of cell membrane permeability and the pH levels (Figure S7), suggesting that decreased pH by acidic PA also contributed to increase the *E. coli* cell membrane permeability. In sum, the increased ROS levels induced by these critical components and low acid pH in PA and its fractions enhanced membrane permeability, thereby promoting conjugative transfer at low amounts.

### 3.6 Increased Intercellular Contact of Bacteria by PA

Direct active intercellular contact is the prerequisite for successful conjugative ARG transfer.^13,49^ EPS, majorly composed of proteins, polysaccharides, extracellular DNA, and lipids,^5^ plays important roles in biofilm formation and bacterial gene transfer by promoting intercellular contact.^38,49^ TEM images showed that PA exposure stimulated the cell-to-cell contact with suspicious surrounding secretions (Figures 5c and S10). Furthermore, EPS content of the mixed *E. coli* strains was measured (Figures 5e and S12a–c). PA exposure at 20 μL significantly increased the contents of proteins and polysaccharides, respectively, by 40 and 13% (Figure S12a,b), and the total EPS contents increased by 29% (Figure S12c). The ratio of proteins to polysaccharides (PN/PS) in EPS, reflecting cell surface hydrophobicity due to the strong hydrophilicity of polysaccharides with rich hydrophilic groups (e.g., carboxyl (−COOH) and hydroxyl (−OH) groups),^49^ also increased in the PA treatments (Figure 5e). Similarly, the three distilled fractions also showed promoted effects on the EPS contents and PN/PS ratio, but the increase levels were lower than those in the PA treatments, consistent with the promotion order of RP4 conjugative transfer (Figure 2a,b). These findings suggested that the PA increased intercellular contact by enhancing the PN/PS ratio to increase cell surface hydrophobicity. Additionally, previous studies showed that some substances such as herbicide (glyphosate, glufosinate, and dicamba), CeO_2_ nanoparticles, and CO_2_ facilitated intercellular contact between donor and recipient strains by regulating EPS composition to enhance ARG conjugation.^38,49^ Therefore, the increased intercellular contact induced by increased cell surface hydrophobicity under PA exposure at low amounts is also another reason for the promoted conjugative transfer of ARGs. However, although the high amount of PA at 40 μL increased EPS content (Figure S12a–c), the conjugation was still inhibited (Figure 2a,b), mainly ascribed to the relatively greater reduction in recipient and donor bacteria number (Figure 2c,d), resulting in the release of proteins or polysaccharides from bacteria. ROS can be used as a signaling molecule via regulating gene expression related to metabolism to stimulate the formation of EPS.^38,71^ The ROS levels had positive correlations with the fold changes of EPS under PA, indicating that the high levels of ROS caused by PA led to high EPS contents (Figure S7). Additionally, bacteria under the exogenous stress, such as low pH or chemicals, could also synthesize and secrete EPS by upregulating the expression of EPS production-related genes,^14,38,67,72^ which act as a barrier to reduce the destruction of the external adverse environment on bacteria themselves.^5,49^ Pearson correlation coefficient analysis showed the significant correlation between the fold changes of EPS or the PN/PS values and the content of 2,6-dimethoxy phenol and 3-methyl-1,2-cyclopentanedione or the pH values (Figure S7). These results suggested that the chemical components of PA, especially 2,6-dimethoxy phenol and 3-methyl-1,2-cyclopentanedione, and the acidic pH by PA contributed to synthesizing and secreting EPS of *E. coli*.

Besides EPS secretion, surface negative charge of bacterial cells, caused by release of protons via ionization of amino acids on cell membrane, is also a key factor determining intercellular contact via electrostatic interactions.^49,73^ Hence, the cell surface zeta potential was further measured (Figures 5e and S12d). Compared with the control, the surface negative charge of *E. coli* cell exposed with PA at 20 μL significantly decreased (Figures 5e and S12d). Similarly, the three fractions also posed decreased effects on the surface negative charge, but the decreased levels were lower than those in the PA treatments (Figures 5e and S12d), consistent with the promoted order on the conjugative transfer (Figure 2a,b). A previous study reported that the zeta potential of cyanobacteria *Microcystis aeruginosa* tended to be 0 under the exposure of a PA synthesized from wheat straw at 800°C, thus removing the electrostatic shield and inducing its flocculation and sedimentation.^74^ These results indicated that the promotion of intercellular contact induced by decreased *E. coli* cell surface charge contributed to the promoted conjugative transfer of RP4 under PA exposure. Unexpectedly, the high amount (40 μL) of PA also decreased the cell surface charge (Figures 5e and S12d), although massive recipient and donor bacteria were inactivated and the conjugation was inhibited (Figure 2c,d). This may be mainly due to the cell surface macromolecules which still could ionize and release the proton regardless of the living or dead cells.

The outer cell membrane is directly interacted with the surrounding environment. Thus, cell surface characteristics (e.g., surface charge and surface hydrophobicity) are easily affected by the surrounding conditions such as nanoparticles,^38^ cations,^66^ and the pH degree.^70,74^ The acidic pH could inhibit ionization of amino acids on cell membranes.^66^ This was supported by the fact that the fold changes of zeta potential values under PA were positively correlated with the pH values or the representative acidic components such as acetic acid and 2-methoxy-phenolx (Figure S7). A previous study showed that soil acidification (pH 5) decreased the surface negative charge of *B. subtilis*, a type of soil-dwelling bacteria beneficial to plant and soil animal growth, by the protonation of its membrane proteins.^66^ These findings suggested that the acidic components of PA decreased the *E. coli* surface negative charge via the protonation of cell membrane proteins, thus promoting the intercellular contact by decreasing intercellular electrostatic repulsion to facilitate the ARG conjugation. Furthermore, soil biofilms, a supracellular structures formed by microorganisms encasing in the self-produced matrix of EPS, represent the predominant microbial lifestyle in soils.^5^ Soil biofilms have a high bacterial density to allow intercellular contact, which are deemed the hotspots of ARG spread and HGT occurrence.^5,75^ However, whether the PA could promote biofilm formation to enhance the ARG conjugative transfer is unclear, which remains to be explored in the future. Furthermore, the changes of microbial communities in the soil environment could also significantly affect the process of HGT in PA-applied soils.^29^ The effect of soil microbial communities on ARG spreading via HGT after applying PA amendments could be further explored with the help of microfluidics and fluorescence in situ detection technology in the future.

## 4 Conclusions and Environmental Implications

**Figure 6:**
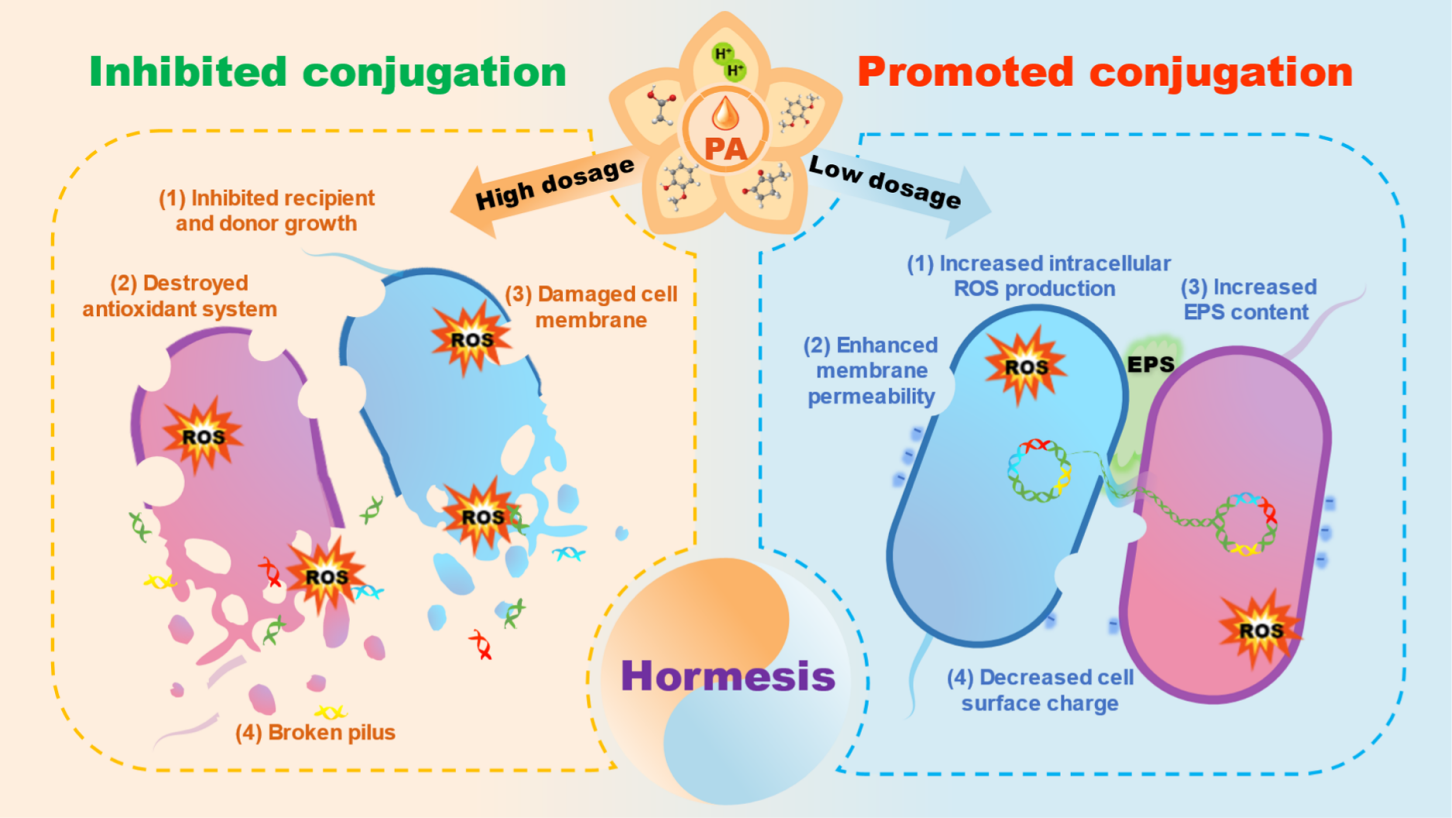
Schematic diagram showing potential mechanisms underlying the hormesis of PA on the conjugative transfer of plasmid RP4 between *E. coli* HB101 and *E. coli* NK5449. The inhibited conjugation at high amounts of PA was mainly due to (1) the inhibited recipient and donor growth, (2) destroyed antioxidant system, (3) damaged cell membrane, and (4) broken pilus, which resulted from the inhibitory levels of antibacterial components of PA such as acetic acid, 2-methoxy-phenolx, 2,6-dimethoxy phenol, and 3-methyl-1,2-cyclopentanedione, as well as the acidic properties (pH ≤ 2.81) of PA. The promoted conjugation at low amounts of PA was mainly ascribed to (1) the increased intracellular ROS production, (2) enhanced cell membrane permeability, (3) increased EPS content, and (4) decreased cell surface charge, which were mainly attributed to the non-lethal stress of representative chemical components of PA and the moderately acidic mating conditions caused by acidic PA.

In this study, we first demonstrated that PA as a soil amendment showed hormesis effects (low-amount promotion and high-amount inhibition) on conjugative transfer of plasmid RP4 within the genus *E. coli*. The inhibited conjugative plasmid RP4 transfer at high amounts (40–100 μL) of PA is ascribed to (Figure 6) (1) the inhibited recipient and donor growth, (2) destroyed antioxidant system, (3) damaged cell membrane, and (4) broken pilus. The inhibited effect mainly resulted from the antibacterial components of PA including acetic acid, 2-methoxy-phenolx, 2,6-dimethoxy phenol, and 3-methyl-1,2-cyclopentanedione, as well as the acidic properties (pH ≤ 2.81) of PA. However, the promoted conjugation at low amounts (10–20 μL) of PA is mainly ascribed to (Figure 6) (1) the increased intracellular ROS production, (2) enhanced cell membrane permeability, (3) increased EPS content, and (4) decreased cell surface charge. The promoted effect was mainly contributed by the non-lethal stress of representative chemical components of PA and the moderately acidic mating conditions caused by strong acidity.

These results provide new insights on the effects of PA amendment on ARG conjugation and convincing evidence for alleviating the spread of soil ARGs using PA amendments at the appropriate application amount to be a practical strategy. Considering the hormesis of PA on the conjugation, our results highlighted the necessity to optimize the amount of soil amendments to alleviate ARG pollution in soils by combating horizontal transfer of ARGs. Moreover, the PA-promoted conjugation also triggered questions regarding the potential risks of PA amendments and other soil amendments having antibacterial activity (e.g., humic acid, lignin material) applied at inappropriate levels in the spread of ARGs via HGT in soil ecosystems, which should be carefully considered. Considering that the representative components of PA (e.g., acids, phenols, and ketones) at non-lethal levels played critical roles in enhanced conjugation, more studies are warranted on optimizing the bacteriostasis of PA by selecting the appropriate feedstock and distilled technology to enhance the efficiency of PA amendments in remediating soil ARG pollution. Furthermore, based on PA-promoted conjugation by enhancing ROS production, membrane permeability, and EPS production, the co-application of PA with some antioxidants (e.g., GSH and thiourea) and EPS scavengers (e.g., metal-based nanomaterials and quorum quenching enzyme) could be feasible strategies to lower the potential risks of PA in facilitating ARG spread through conjugation. However, only one woody waste-derived PA was selected in the present study to investigate its effect on ARG conjugation among intrageneric bacteria using a pure culture model; further studies are warranted to examine the effects of more types of PA amendments on intrageneric and intergeneric conjugative transfer or other HGT routines (e.g., transformation and transduction) among the microbial communities in practical soil ecosystems.

## Supporting Information Available

Additional information on detailed experimental methods, material characterization, transconjugant number, conjugative transfer frequency, recipient and donor number, evidence of transconjugant production, bacterial activity, Pearson correlation analysis, and contents of EPS and zeta potential (PDF)

## References

[ref1] Crofts T.; Gasparrini A.; Dantas G. Next-generation approaches to understand and combat the antibiotic resistome. Nat. Rev. Microbiol. 2017, 15, 422–434. 10.1038/nrmicro.2017.28. 28392565 PMC5681478

[ref2] Yuan L.; Wang Y.; Zhang L.; Palomo A.; Zhou J.; Smets B.; Bürgmann H.; Ju F. Pathogenic and indigenous denitrifying bacteria are transcriptionally active and key multi-antibiotic-resistant players in wastewater treatment plants. Environ. Sci. Technol. 2021, 55, 10862–10874. 10.1021/acs.est.1c02483. 34282905

[ref3] Lu J.; Guo J. Disinfection spreads antimicrobial resistance. Science 2021, 371, 474 10.1126/science.abg4380. 33510019

[ref4] Birmes L.; Freese H.; Petersen J. RepC_soli: a novel promiscuous plasmid type of Rhodobacteraceae mediates horizontal transfer of antibiotic resistances in the ocean. Environ. Microbiol. 2021, 23, 5395–5411. 10.1111/1462-2920.15380. 33393148

[ref5] Wu S.; Wu Y.; Cao B.; Huang Q.; Cai P. An invisible workforce in soil: the neglected role of soil biofilms in conjugative transfer of antibiotic resistance genes. Crit. Rev. Environ. Sci. Technol. 2021, 52, 2720–2748. 10.1080/10643389.2021.1892015.

[ref6] Yan Z.-Z.; Chen Q.; Li C.; Nguyen B.; Zhu Y.; He J.; Hu H. Biotic and abiotic factors distinctly drive contrasting biogeographic patterns between phyllosphere and soil resistomes in natural ecosystems. ISME Commun. 2021, 1, 1–9. 10.1038/s43705-021-00012-4. PMC9645249 36721011

[ref7] Chen Z.; Fu Q.; Wen Q.; Wu Y.; Bao H.; Guo J. Microbial community competition rather than high-temperature predominates ARGs elimination in swine manure composting. J. Hazard. Mater. 2022, 423, 127149 10.1016/j.jhazmat.2021.127149. 34530271

[ref8] Hilaire S. S.; Chen C.; Pan Z.; Radolinski J.; Stewart R. D.; Maguire R. O.; Xia K. Subsurface manure injection reduces surface transport of antibiotic resistance genes but may create antibiotic resistance hotspots in soils. Environ. Sci. Technol. 2022, 56, 14972–14981. 10.1021/acs.est.2c00981. 35839145

[ref9] Marano R.; Gupta C.; Cozer T.; Jurkevitch E.; Cytryn E. Hidden Resistome: Enrichment reveals the presence of clinically relevant antibiotic resistance determinants in treated wastewater-irrigated soils. Environ. Sci. Technol. 2021, 55, 6814–6827. 10.1021/acs.est.1c00612. 33904706

[ref10] Zheng H.; Feng N.; Yang T.; Shi M.; Wang X.; Zhang Q.; Zhao J.; Li F.; Sun K.; Xing B. Individual and combined applications of biochar and pyroligneous acid mitigate dissemination of antibiotic resistance genes in agricultural soil. Sci. Total Environ. 2021, 796, 148962 10.1016/j.scitotenv.2021.148962. 34271377

[ref11] Fu Y.; Jia M.; Wang F.; Wang Z.; Mei Z.; Bian Y.; Jiang X.; Virta M.; Tiedje J. Strategy for mitigating antibiotic resistance by biochar and hyperaccumulators in cadmium and oxytetracycline co-contaminated soil. Environ. Sci. Technol. 2021, 55, 16369–16378. 10.1021/acs.est.1c03434. 34695355

[ref12] Xing Y.; Kang X.; Zhang S.; Men Y. Specific phenotypic, genomic, and fitness evolutionary trajectories toward streptomycin resistance induced by pesticide co-stressors in Escherichia coli. ISME Commun. 2021, 1, 1–11. 10.1038/s43705-021-00041-z. PMC9723568 37938677

[ref13] Brito I. Examining horizontal gene transfer in microbial communities. Nat. Rev. Microbiol. 2021, 19, 442–453. 10.1038/s41579-021-00534-7. 33846600

[ref14] Li X.; Wen C.; Liu C.; Lu S.; Xu Z.; Yang Q.; Chen Z.; Liao H.; Zhou S. Herbicide promotes the conjugative transfer of multi-resistance genes by facilitating cellular contact and plasmid transfer. J. Environ. Sci. 2022, 115, 363–373. 10.1016/j.jes.2021.08.006. 34969463

[ref15] Sun Y.; Snow D.; Walia H.; Li X. Transmission routes of the microbiome and resistome from manure to soil and lettuce. Environ. Sci. Technol. 2021, 55, 11102–11112. 10.1021/acs.est.1c02985. 34323079

[ref16] Zhu D.; Xiang Q.; Yang X.; Ke X.; O’Connor P.; Zhu Y. Trophic transfer of antibiotic resistance genes in a soil detritus food chain. Environ. Sci. Technol. 2019, 53, 7770–7781. 10.1021/acs.est.9b00214. 31244079

[ref17] Xu L.; Gu J.; Wang X.; Song Z.; Jiang H.; Li N.; Lei L.; Xie J.; Hu T.; Ding Q.; Sun Y. Risk of horizontal transfer of intracellular, extracellular, and bacteriophage antibiotic resistance genes during anaerobic digestion of cow manure. Bioresour. Technol. 2022, 351, 127007 10.1016/j.biortech.2022.127007. 35304254

[ref18] Zammit I.; Marano R.; Vaiano V.; Cytryn E.; Rizzo L. Changes in antibiotic resistance gene levels in soil after irrigation with treated wastewater: a comparison between heterogeneous photocatalysis and chlorination. Environ. Sci. Technol. 2020, 54, 7677–7686. 10.1021/acs.est.0c01565. 32412248 PMC8007107

[ref19] Zhu D.; Delgado-Baquerizo M.; Su J.; Ding J.; Li H.; Gillings M.; Penuelas J.; Zhu Y. Deciphering potential roles of earthworms in mitigation of antibiotic resistance in the soils from diverse ecosystems. Environ. Sci. Technol. 2021, 55, 7445–7455. 10.1021/acs.est.1c00811. 33977709

[ref20] Zhu D.; Ding J.; Yin Y.; Ke X.; O’Connor P.; Zhu Y. Effects of earthworms on the microbiomes and antibiotic resistomes of detritus fauna and phyllospheres. Environ. Sci. Technol. 2020, 54, 6000–6008. 10.1021/acs.est.9b04500. 32352284

[ref21] Chen M.; An X.; Liao H.; Yang K.; Su J.; Zhu Y. Viral community and virus-associated antibiotic resistance genes in soils amended with organic fertilizers. Environ. Sci. Technol. 2021, 55, 13881–13890. 10.1021/acs.est.1c03847. 34596377

[ref22] Ye M.; Sun M.; Feng Y.; Li X.; Schwab A.; Wan J.; Liu M.; Tian D.; Liu K.; Wu J.; Jiang X. Calcined eggshell waste for mitigating soil antibiotic-resistant bacteria/antibiotic resistance gene dissemination and accumulation in bell pepper. J. Agric. Food Chem. 2016, 64, 5446–5453. 10.1021/acs.jafc.6b00866. 27333280

[ref23] Li Y.; Deng M.; Wang X.; Wang Y.; Li J.; Xia S.; Zhao J. In-situ remediation of oxytetracycline and Cr(VI) co-contaminated soil and groundwater by using blast furnace slag-supported nanosized Fe^0^/FeS_x_. Chem. Eng. J. 2021, 412, 128706 10.1016/j.cej.2021.128706.

[ref24] Mohammadi-Aragh M.; Stokes C.; Street J.; Linhoss J. Effects of loblolly pine biochar and wood vinegar on poultry litter nutrients and microbial abundance. Animals 2021, 11, 2209 10.3390/ani11082209. 34438667 PMC8388362

[ref25] Zhang Y.; Wang X.; Liu B.; Liu Q.; Zheng H.; You X.; Sun K.; Luo X.; Li F. Comparative study of individual and co-application of biochar and wood vinegar on blueberry fruit yield and nutritional quality. Chemosphere 2020, 246, 125699 10.1016/j.chemosphere.2019.125699. 31884234

[ref26] Soares W.; Lira G.; Santos C.; Dias G.; Pimenta A.; Pereira A.; Benício L.; Rodrigues G.; Amora S.; Alves N.; Feijó F. Pyroligneous acid from Mimosa tenuiflora and Eucalyptus urograndis as an antimicrobial in dairy goats. J. Appl. Microbiol. 2021, 131, 604–614. 10.1111/jam.14977. 33342017

[ref27] Zhang C.; Luo S.; Xu F.; Bu Q.; Qiu L. Chemical characteristics and antimicrobial performance of wood vinegar produced from pyrolysis of polyploidy mulberry branches. J. Biobased Mater. Bio. 2019, 13, 812–819. 10.1166/jbmb.2019.1923.

[ref28] Korkalo P.; Hagner M.; Jänis J.; Mäkinen M.; Kaseva J.; Lassi U.; Rasa K.; Jyske T. Pyroligneous acids of differently pretreated hybrid aspen biomass: herbicide and fungicide performance. Front. Chem. 2021, 9, 821806 10.3389/fchem.2021.821806. 35211460 PMC8861299

[ref29] Zheng H.; Wang R.; Zhang Q.; Zhao J.; Li F.; Luo X.; Xing B. Pyroligneous acid mitigated dissemination of antibiotic resistance genes in soil. Environ. Int. 2020, 145, 106158 10.1016/j.envint.2020.106158. 33038622

[ref30] Guo H.; Gu J.; Wang X.; Yu J.; Nasir M.; Peng H.; Zhang R.; Hu T.; Wang Q.; Ma J. Responses of antibiotic and heavy metal resistance genes to bamboo charcoal and bamboo vinegar during aerobic composting. Environ. Pollut. 2019, 252, 1097–1105. 10.1016/j.envpol.2019.05.014. 31252107

[ref31] Arnold B.; Huang I.; Hanage W. Horizontal gene transfer and adaptive evolution in bacteria. Nat. Rev. Microbiol. 2021, 20, 206–218. 10.1038/s41579-021-00650-4. 34773098

[ref32] Lee I.; Eldakar O.; Gogarten J.; Andam C. Bacterial cooperation through horizontal gene transfer. Trends. Ecol. Evol. 2022, 37, 223–232. 10.1016/j.tree.2021.11.006. 34815098

[ref33] Ye M.; Zhang Z.; Sun M.; Shi Y. Dynamics, gene transfer, and ecological function of intracellular and extracellular DNA in environmental microbiome. iMeta 2022, 1, e34 10.1002/imt2.34. PMC10989830 38868707

[ref34] He Y.; Yuan Q.; Mathieu J.; Stadler L.; Senehi N.; Sun R.; Alvarez P. Antibiotic resistance genes from livestock waste: occurrence, dissemination, and treatment. npj Clean Water 2020, 3, 1–11. 10.1038/s41545-020-0051-0.

[ref35] Liu G.; Bogaj K.; Bortolaia V.; Olsen J.; Thomsen L. Antibiotic-induced, increased conjugative transfer is common to diverse naturally occurring ESBL plasmids in *Escherichia coli*. Front. Microbiol. 2019, 10, 2119 10.3389/fmicb.2019.02119. 31552012 PMC6747055

[ref36] Cen T.; Zhang X.; Xie S.; Li D. Preservatives accelerate the horizontal transfer of plasmid-mediated antimicrobial resistance genes via differential mechanisms. Environ. Int. 2020, 138, 105544 10.1016/j.envint.2020.105544. 32172042

[ref37] Li Z.; Gao J.; Guo Y.; Cui Y.; Wang Y.; Duan W.; Wu Z. Enhancement of antibiotic resistance dissemination by artificial sweetener acesulfame potassium: insights from cell membrane, enzyme, energy supply and transcriptomics. J. Hazard. Mater. 2021, 422, 126942 10.1016/j.jhazmat.2021.126942. 34449343

[ref38] Yu K.; Chen F.; Yue L.; Luo Y.; Wang Z.; Xing B. CeO_2_ nanoparticles regulate the propagation of antibiotic resistance genes by altering cellular contact and plasmid transfer. Environ. Sci. Technol. 2020, 64, 5446–5453. 10.1021/acs.est.0c01870. 32806911

[ref39] Jutkina J.; Marathe N.; Flach C.; Larsson D. Antibiotics and common antibacterial biocides stimulate horizontal transfer of resistance at low concentrations. Sci. Total Environ. 2018, 616, 172–178. 10.1016/j.scitotenv.2017.10.312. 29112840

[ref40] Liu X.; Wang D.; Tang J.; Liu F.; Wang L. Effect of dissolved biochar on the transfer of antibiotic resistance genes between bacteria. Environ. Pollut. 2021, 288, 117718 10.1016/j.envpol.2021.117718. 34274650

[ref41] Ghidotti M.; Fabbri D.; Mašek O.; Mackay C.; Montalti M.; Hornung A. Source and biological response of biochar organic compounds released into water; relationships with bio-oil composition and carbonization degree. Environ. Sci. Technol. 2017, 51, 6580–6589. 10.1021/acs.est.7b00520. 28437609

[ref42] Li R.; Narita R.; Ouda R.; Kimura C.; Nishimura H.; Yatagai M.; Fujita T.; Watanabe T. Structure-dependent antiviral activity of catechol derivatives in pyroligneous acid against the encephalomycarditis virus. RSC Adv. 2018, 8, 35888–35896. 10.1039/c8ra07096b. 35558500 PMC9088284

[ref43] Suresh G.; Pakdel H.; Rouissi T.; Brar S. K.; Fliss I.; Roy C. In vitro evaluation of antimicrobial efficacy of pyroligneous acid from softwood mixture. Bi. Res. Innovation 2019, 3, 47–53. 10.1016/j.biori.2019.02.004.

[ref44] Ma X.; Zhang X.; Xia J.; Sun H.; Zhang X.; Ye L. Phenolic compounds promote the horizontal transfer of antibiotic resistance genes in activated sludge. Sci. Total Environ. 2021, 800, 149549 10.1016/j.scitotenv.2021.149549. 34392203

[ref45] Fierer N. Embracing the unknown: disentangling the complexities of the soil microbiome. Nat. Rev. Microbiol. 2017, 15, 579–590. 10.1038/nrmicro.2017.87. 28824177

[ref46] Lu J.; Wang Y.; Li J.; Mao L.; Nguyen S.; Duarte T.; Coin L.; Bond P.; Yuan Z.; Guo J. Triclosan at environmentally relevant concentrations promotes horizontal transfer of multidrug resistance genes within and across bacterial genera. Environ. Int. 2018, 121, 1217–1226. 10.1016/j.envint.2018.10.040. 30389380

[ref47] Li H.; Kang Z.; Jiang E.; Song R.; Zhang Y.; Qu G.; Wang T.; Jia H.; Zhu L. Plasma induced efficient removal of antibiotic-resistant *Escherichia coli* and antibiotic resistance genes, and inhibition of gene transfer by conjugation. J. Hazard. Mater. 2021, 419, 126465 10.1016/j.jhazmat.2021.126465. 34214852

[ref48] Tashiro T.; Yoshimura F. A neo-logistic model for the growth of bacteria. Physica A 2019, 525, 199–215. 10.1016/j.physa.2019.03.049.

[ref49] Liao J.; Huang H.; Chen Y. CO_2_ promotes the conjugative transfer of multiresistance genes by facilitating cellular contact and plasmid transfer. Environ. Int. 2019, 129, 333–342. 10.1016/j.envint.2019.05.060. 31150975

[ref50] Wang D.; Saleh N.; Byro A.; Zepp R.; Sahle-Demessie E.; Luxton T.; Ho K.; Burgess R.; Flury M.; White J.; Su C. Nano-enabled pesticides for sustainable agriculture and global food security. Nat. Nanotechnol. 2022, 17, 347–360. 10.1038/s41565-022-01082-8. 35332293 PMC9774002

[ref51] Guo Y.; Gao J.; Cui Y.; Wang Z.; Li Z.; Duan W.; Wang Y.; Wu Z. Chloroxylenol at environmental concentrations can promote conjugative transfer of antibiotic resistance genes by multiple mechanisms. Sci. Total Environ. 2022, 816, 151599 10.1016/j.scitotenv.2021.151599. 34774958

[ref52] Huang H.; Liao J.; Zheng X.; Chen Y.; Ren H. Low-level free nitrous acid efficiently inhibits the conjugative transfer of antibiotic resistance by altering intracellular ions and disabling transfer apparatus. Water Res. 2019, 158, 383–391. 10.1016/j.watres.2019.04.046. 31059932

[ref53] Pisoschi A.; Pop A.; Georgescu C.; Turcuş V.; Olah N.; Mathe E. An overview of natural antimicrobials role in food. Eur. J. Med. Chem. 2018, 143, 922–935. 10.1016/j.ejmech.2017.11.095. 29227932

[ref54] Jong M.; Harwood C.; Blackburn A.; Snape J.; Graham D. Impact of redox conditions on antibiotic resistance conjugative gene transfer frequency and plasmid fate in wastewater ecosystems. Environ. Sci. Technol. 2020, 54, 14984–14993. 10.1021/acs.est.0c03714. 33191749

[ref55] Huang J.; Zhu J.; Liu S.; Luo Y.; Zhao R.; Guo F.; Li B. Estuarine salinity gradient governs sedimentary bacterial community but not antibiotic resistance gene profile. Sci. Total Environ. 2022, 806, 151390 10.1016/j.scitotenv.2021.151390. 34740654

[ref56] Qiu Z.; Yu Y.; Chen Z.; Jin M.; Yang D.; Zhao Z.; Wang J.; Shen Z.; Wang X.; Qian D.; Huang A.; Zhang B.; Li J. Nanoalumina promotes the horizontal transfer of multiresistance genes mediated by plasmids across genera. Proc. Natl. Acad. Sci. USA. 2012, 109, 4944–4949. 10.1073/pnas.1107254109. 22411796 PMC3323979

[ref57] Huang H.; Chen Y.; Zheng X.; Su Y.; Wan R.; Yang S. Distribution of tetracycline resistance genes in anaerobic treatment of waste sludge: the role of pH in regulating tetracycline resistant bacteria and horizontal gene transfer. Bioresour. Technol. 2016, 218, 1284–1289. 10.1016/j.biortech.2016.07.097. 27485281

[ref58] Li S.; Yao Q.; Liu J.; Yu Z.; Li Y.; Jin J.; Liu X.; Wang G. Liming mitigates the spread of antibiotic resistance genes in an acid black soil. Sci. Total Environ. 2022, 817, 152971 10.1016/j.scitotenv.2022.152971. 35016930

[ref59] Zhang H.; Jin W.; Dong C.; Liu C. Conformational change of *Escherichia coli* alkaline phosphatase in various media. Chinese J. Anal. Chem. 2000, 28, 59–60.

[ref60] Nagachinta S.; Chen J. Transfer of class 1 integron-mediated antibiotic resistance genes from shiga toxin-producing Escherichia coli to a susceptible *E. coli* K-12 strain in storm water and bovine feces. Appl. Environ. Microbiol. 2008, 74, 5063–5067. 10.1128/aem.00517-08. 18552189 PMC2519284

[ref61] Yuan Q.; Sun R.; Yu P.; Cheng Y.; Wu W.; Bao J.; Alvarez P. UV-aging of microplastics increases proximal ARG donor-recipient adsorption and leaching of chemicals that synergistically enhance antibiotic resistance propagation. J. Hazard. Mater. 2022, 427, 127895 10.1016/j.jhazmat.2021.127895. 34844806

[ref62] Lin H.; Sun W.; Yu Q.; Ma J. Acidic conditions enhance the removal of sulfonamide antibiotics and antibiotic resistance determinants in swine manure. Environ. Pollut. 2020, 263, 114439 10.1016/j.envpol.2020.114439. 32302890

[ref63] Van Acker H.; Coenye T. The role of reactive oxygen species in antibiotic-mediated killing of bacteria. Trends Microbiol. 2017, 25, 456–466. 10.1016/j.tim.2016.12.008. 28089288

[ref64] Mantilla-Calderon D.; Plewa M.; Michoud G.; Fodelianakis S.; Daffonchio D.; Hong P. Water disinfection byproducts increase natural transformation rates of environmental DNA in *Acinetobacter baylyi* ADP1. Environ. Sci. Technol. 2019, 53, 6520–6528. 10.1021/acs.est.9b00692. 31050420

[ref65] Foreman B.; Tarloff J. Contribution of reactive oxygen species to *para*-aminophenol toxicity in LLC-PK1 cells. Toxicol. Appl. Pharmacol. 2008, 230, 144–149. 10.1016/j.taap.2008.02.014. 18396305 PMC2491647

[ref66] Li J.; Zheng T.; Liu C. Soil acidification enhancing the growth and metabolism inhibition of PFOS and Cr(VI) to bacteria involving oxidative stress and cell permeability. Environ. Pollut. 2021, 275, 116650 10.1016/j.envpol.2021.116650. 33581635

[ref67] Wu S.; Mi T.; Zhen Y.; Yu K.; Wang F.; Yu Z. A rise in ROS and EPS production: new insights into the trichodesmium erythraeum response to ocean acidification. J. Phycol. 2021, 57, 172–182. 10.1111/jpy.13075. 32975309

[ref68] Nagar E.; Zilberman S.; Sendersky E.; Simkovsky R.; Shimoni E.; Gershtein D.; Herzberg M.; Golden S. S.; Schwarz R. Type 4 pili are dispensable for biofilm development in the cyanobacterium *Synechococcus elongatus*. Environ. Microbiol. 2017, 19, 2862–2872. 10.1111/1462-2920.13814. 28585390

[ref69] Yoon Y.; Lee H.; Lee S.; Kim S.; Choi K. Membrane fluidity-related adaptive response mechanisms of foodborne bacterial pathogens under environmental stresses. Food Res. Int. 2015, 72, 25–36. 10.1016/j.foodres.2015.03.016.

[ref70] Li Y.; Yan P.; Lei Q.; Li B.; Sun Y.; Li S.; Lei H.; Xie N. Metabolic adaptability shifts of cell membrane fatty acids of *Komagataeibacter hansenii* HDM1-3 improve acid stress resistance and survival in acidic environments. J. Ind. Microbiol. Biotechnol. 2019, 46, 1491–1503. 10.1007/s10295-019-02225-y. 31512094

[ref71] Han P.; Shen S.; Guo R.; Zhao D.; Lin Y.; Jia S.; Yan R.; Wu Y. ROS is a factor regulating the increased polysaccharide production by light quality in the edible cyanobacterium nostoc flagelliforme. J. Agric. Food Chem. 2019, 67, 2235–2244. 10.1021/acs.jafc.8b06176. 30724068

[ref72] Qiu L.; Wu J.; Du W.; Nafees M.; Yin Y.; Ji R.; Banwart S.; Guo H. Response of soil bacterial communities to sulfadiazine present in manure: protection and adaptation mechanisms of extracellular polymeric substances. J. Hazard. Mater. 2020, 408, 124887 10.1016/j.jhazmat.2020.124887. 33387717

[ref73] Li H.; Song R.; Wang Y.; Zhong R.; Zhang Y.; Zhou J.; Wang T.; Jia H.; Zhu L. Inhibited conjugative transfer of antibiotic resistance genes in antibiotic resistant bacteria by surface plasma. Water Res. 2021, 204, 117630 10.1016/j.watres.2021.117630. 34536683

[ref74] Zhu Y.; Cheng S.; Wang P.; Chen H.; Zhang X.; Liu L.; Li X.; Ding Y. A possible environmental-friendly removal of *Microcystis aeruginosa* by using pyroligneous acid. Ecotoxicol. Environ. Saf. 2020, 205, 111159 10.1016/j.ecoenv.2020.111159. 32829212

[ref75] Olsen I. Biofilm-specific antibiotic tolerance and resistance. Eur. J. Clin. Microbiol. Infect. Dis. 2015, 34, 877–886. 10.1007/s10096-015-2323-z. 25630538

