# Supplementary material for: Hormetic Effect of Pyroligneous Acids on Conjugative Transfer of Plasmid-mediated Multi-antibiotic Resistance Genes within Bacterial Genus
Source: ACS Environ Au. 2022 Dec 22;3(2):105–20. doi: 10.1021/acsenvironau.2c00056 (PMC10125354; doi:10.1021/acsenvironau.2c00056)
Supplement: Supplementary file 1 — vg2c00056_si_001.pdf [file vg2c00056_si_001.pdf]

Supplementary Information

Hormetic effect of pyroligneous acids on conjugative transfer of plasmid-mediated multi-antibiotic resistance genes within bacterial genus

Mengying Shao[a,b], Liuqingqing Liu[a,b], Bingjie Liu[a,c], Hao Zheng[a,b,d,*], Wei Meng[a], Yifan Liu[a], Xiao Zhang[a], Xiaohan Ma[a,b], Cuizhu Sun[a,b], Xianxiang Luo[a,b,d], Fengmin Li[a,b,d], Baoshan Xing[e,*]

[a]*Institute of Coastal Environmental Pollution Control, College of Environmental Science and Engineering, Ministry of Education Key Laboratory of Marine Environment and Ecology, Frontiers Science Center for Deep Ocean Multispheres* and *Earth System, Ocean University of China, Qingdao 266100, China*

[b]*Marine Ecology and Environmental Science Laboratory, Qingdao National Laboratory for Marine Science and Technology, Qingdao 266071, China*

[c]*South China Institute of Environmental Sciences, Ministry of Ecology and Environment, Guangzhou 510535, China*

[d]*Sanya Oceanographic Institution, Ocean University of China, Sanya 572000, China*

[e]*Stockbridge School of Agriculture, University of Massachusetts, Amherst, Massachusetts 01003, United States*

*Corresponding authors: zhenghao2013@ouc.edu.cn (Dr. Zheng); bx@umass.edu (Dr. Xing)

Name of the journal: ***ACS Environmental Au***

Date of the document prepared: *Dec 13, 2022*

Number of pages: *30*

Number of tables: *8*

Number of figures: *12*

Table of Contents

Text S1. Plasmid extraction and gel electrophoresis.

Text S2. Determination of antibacterial activity of PA and its fractions.

Text S3. Measurement of minimum inhibitory concentrations (MICs).

Text S4. Measurement of reactive oxygen species (ROS).

Text S5. Microstructure observation of mixed bacteria strains by transmission electron microscope (TEM) and measurement of cell membrane permeability.

Text S6. Measurement of extracellular polymeric substances (EPS).

Text S7. Inhibited bacterial activity by PA and the fractions.

Table S1. Summary of the applied amounts of PA in reported studies.

Table S2. Minimum inhibitory concentrations (MICs) of PA, the three distilled fractions, and the four representative components.

Table S3. The transconjugants number, conjugative transfer frequency, recipient and donor number in a 30-mL mating system under exposure of PA.

Table S4. The transconjugants number, conjugative transfer frequency, recipient and donor number in a 30-mL mating system under exposure of PA and its three fractions.

Table S5. The transconjugants number, conjugative transfer frequency, recipient and donor number in a 30-mL mating system under exposure of four representative components.

Table S6. The pHs of three mating systems with different treatments.

Table S7. The pHs of four representative components with different concentrations in Milli-Q water.

Table S8. Fitting parameters of growth curves of *E. coli* under PA and its fractions by logistic growth mode.

Figure S1. Properties of PA and its distilled fractions as previous reported.

Figure S2. Effect of ROS scavenger glutathione (GSH) at different concentrations on the growth of (a) recipient *E. coli* NK5449 and (b) donor *E. coli* HB101.

Figure S3. Evidence of transconjugants production under PA and its fractions exposure.

Figure S4. LCSM images of donor *E. coli* HB101 (a) and recipient *E. coli* NK5449 (b) exposed with 0, 10 and 40 μL PA or its fractions in a 30-mL mating system.

Figure S5. Effects of PA and its fractions on the growth of *E. coli* bacterial strains.

Figure S6. Effects of four representative components of PA on the strain number of the recipient *E. coli* NK5449 and donor *E. coli* HB101.

Figure S7. Pearson correlation analysis for exploring the relationships between fold changes of transconjugant number (FTN), fold changes of donor number (FDN), fold changes of recipient number (FRN), acetic acid, 2-methoxy-phenolx, 2, 6-dimethoxy phenol, and 3-methyl-1, 2-cyclopentanedione contents of PA, pHs of mating systems, ROS, cell membrane permeability, EPS, PN/PS, and zeta potential levels of recipient and donor strains.

Figure S8. Effects of pH in a 30-mL mating system exposed with PA and its distilled fractions at 40 μL (a, b) and 20 μL (c, d) on the growth of recipient NK5449 and donor *E. coli* HB101.

Figure S9. Effect of ROS scavenger glutathione (GSH) on the number of (a) recipient *E. coli* NK5449 and (b) donor *E. coli* HB101 under PA exposure.

Figure S10. TEM images of the mixed donor *E. coli* HB101 and recipient *E. coli* NK5449 exposed to (a) PA and (b) its distilled fractions.

Figure S11. Fold changes in cell membrane permeability of the mixed donor *E. coli* HB101 and recipient *E. coli* NK5449 treated with ROS scavenger GSH (300 μmol/L) under PA exposure.

Figure S12. Effects of PA and its distilled on the contents of proteins (a), polysaccharides (b), EPS (c), and the zeta potential (d) in the mixed donor *E. coli* HB101 and recipient *E. coli* NK5449.

Text S1. Plasmid extraction and gel electrophoresis

To confirm the successful transfer of RP4 plasmids from donor *E. coli* HB101 to recipient *E. coli* NK5449, agarose gel electrophoresis was applied to verify the presence of RP4 plasmids in the transconjugants.[1] Briefly, five donors and transconjugants colonies were stochastically picked out from the donor-selective plates and transconjugant-selective plates respectively, and incubated at 37°C for 16 h.[2] The RP4 plasmids in donors and transconjugants were extracted by Plasmid Extraction Kit (Insight Exbio Technology, China), following the manufacturer's instructions. Then 1 μL extracted plasmid solution was mixed with 1 μL gel loading buffer and 5 μL sterilized Milli-Q water. Subsequently, the mixed samples were loaded onto 1% agarose gel with 4S GelRed nucleic acid stain (Life Technologies, USA) to electrophoresis. Meanwhile, 5 μL DNA marker with size of 100~5000 bp (Life Technologies, USA) as reference was loaded onto the first hole of the gel. The gel with mixed samples and DNA Marker sample was conducted in 1 × TAE buffer solution at 110 V for 30 min, and then the gel was put into a Tanon 1600 imaging system (Azure Biosystems C150, U.S.) to get visualized DNA bands.

Text S2. Determination of antibacterial activity of PA and its fractions

To verify the effect of PA and its fractions on the growth of donor, recipient, and transconjugant strains, three different assays were employed. First, a LB-agar plate counting method was used to examine the bacterial activity exposed to different amounts of PA and its fractions.[3] After cultivated in 100 mL LB broth medium at 37°C and 200 rpm for 12 h, the donor and recipient bacteria pellets were obtained by centrifuging the culture at 4000 rpm and 25°C for 10 min to remove the supernatant, and then the bacteria pellets were washed twice with phosphate buffer saline (PBS, pH 7.2) and resuspended in LB liquid medium to obtain the desired bacterial concentration (3×10^8 CFU/mL). Then, 150 μL donor and 150 μL recipient bacteria suspensions were added into 30 mL LB liquid medium containing different amounts of PA (0, 10, 20, 40, 60, 80, and 100 μL) and the three distilled fractions (0, 10, 20, and 40 μL). Following incubation at 37°C and 200 rpm for 18 h, the mixtures were appropriately diluted with sterile 0.9% NaCl based on the expected number of colonies. Then 100 μL of the diluted mixtures was plated on LB agar plates containing the corresponding antibiotics to select the donor (Amp^R, Km^R, Tet^R), recipient (Rif^R) or transconjugant (Amp^R, Km^R, Tet^R, and Rif^R) and then incubated at 37 °C for 24 h to obtain viable counts of donor, recipient, and transconjugant.

Second, a LIVE/DEAD bacterial viability assay method was employed to observe the dead and live bacterial cells exposed to different amounts of PA and its fractions using an A1 laser confocal scanning microscope (LCSM, Nikon, Japan).[4] Briefly, after overnight incubation, the donor and recipient were centrifuged at 4000 rpm for 10 min to remove the supernatant, then the donor and recipient pellets were resuspended with LB liquid medium and adjusted to approximately 10^8 CFU/mL. PA (10, 40 μL) and its fractions (10, 40 μL) were added into the bacterial suspension and cultured at 37°C and 200 rpm for 2 h. After the incubation, the donor and recipient bacteria solution were centrifuged at 4000 rpm for 10 min and resuspended with 20 mL 0.85% NaCl, and were incubated at 25°C for 1 h and gently shaken every 15 min. Then the bacteria pellets were collected by centrifuging at 4000 rpm for 10 min to remove the supernatant, and then were resuspended with 10 mL 0.85% NaCl. Subsequently, 1.5 μL SYTO 9 green fluorescent nucleic acid stain and PI dye were respectively added into 1 mL bacterial suspension, and then incubate at 25°C for 15 min and mix it every 5 min in the dark. After staining, 10 μL bacterial suspension was placed on the glass slide. The living bacteria carried green fluorescence was observed at 500 nm with excitation at 480 nm and the dead bacteria carried red fluorescence was observed at 635 nm with excitation at 490 nm using the LCSM.

The experiment was conducted in biological triplicates along with blank control without PA or its fractions addition.

Third, the growth curves of these three bacterial strains were measured using a UV-visible spectrophotometer (UV-3300PC, MAPADA instruments Co., Ltd., China) China) in the presence and absences of PA and its fractions.[5] The donor, recipient, and transconjugant bacterial suspension (300 μL) was separately cultured in LB broth medium added with different amounts of PA or its fractions (10, 20, 40 μL) at 37°C for 18 h. During the incubation, the optical density at 600 nm (OD_{600}) was measured every 2 h using the UV-visible spectrophotometer to obtain the bacterial growth curve. The bacteria growth curves were fitted using logistic growth model ($y = a/(1 + \exp(-k*(x-x_c)))$) with Origin 2022. The values of R^2, fitting coefficient, were greater than 0.900 (Table S4), according with the sigmoidal characteristic, which indicated that the growth of donor, recipient and transconjugant strains would be limited by nutrition, space or other environmental constraints. Each test was carried out in triplicate at least.

Text S3. Measurement of minimum inhibitory concentrations (MICs)

The 10%, 50% and 90% minimum inhibitory concentration (MIC_{10}, MIC_{50}, and MIC_{90}), representing the amounts of PA, the three fractions (F1, F2, and F3) or four representative components (i.e., acetic acid, guaiacol, 2,6-dimethoxyphenol, and 3-methyl-1,2-cyclopentadione) that caused 10%, 50%, and 90% growth inhibition of the recipient *E. coli* NK5449 and donor *E. coli* HB101, were detected based on the EUCAST clinical breakpoints methods.[6] In detail, the strains were inoculated overnight and adjusted to approximately 10^8 CFU/mL using LB broth medium. Then 300 μL of cell suspension and 30 mL LB liquid medium were added into sterile conical flask containing different amounts of PA or its three fractions (10, 20, and 40 μL), and 5 μL of cell suspension and 130 μL of fresh LB media were added into each well of the 96-well plates containing 15 μL of different amounts of acetic acid (0.00625, 0.0125, 0.025, 0.05, 0.1, 0.2, 0.4 mg/mL), 2-methoxy-phenolx (0.01, 0.02, 0.04, 0.08, 0.16, 0.32, 0.64 mg/mL), 2,6-dimethoxyphenol (0.015625, 0.03125, 0.0625, 0.125, 0.25, 0.5, 1 mg/mL), or 3-methyl-1,2-cyclopentadione (0.015625, 0.03125, 0.0625, 0.125, 0.25, 0.5, 1 mg/mL), respectively. The sterilized Milli-Q water was set as the blank control. After the plates were incubated at 37°C for 18 h, the optical density at 600 nm (OD_{600}) was measured by UV-visible spectrophotometer (UV-3300PC, MAPADA instruments Co., Ltd., China) and microplate reader (1500, Thermo, USA). All the experiments were conducted with biological triplicates. Probit model was used to calculate the MIC_{10}, MIC_{50}, and MIC_{90} by SPSS 20.0.[7]

Text S4. Measurement of reactive oxygen species (ROS)

The intracellular ROS level of the bacteria was measured using a 2’,7’-dichlorofluorescein diacetate (DCFDA) cellular ROS detection assay kit (Beyotime, China).[1] Briefly, the mixed bacterial concentration of donor *E. coli* HB101 and recipient *E. coli* NK5449 (1:1, v/v) was adjusted to 10^6 CFU/mL with PBS (pH 7.2), and then incubated with 5 mL of 10 μM DCFH-DA at 37°C for 30 min in the dark. Subsequently, the cell suspension was washed with PBS three times to remove the unbound extracellular DCFH-DA. Then different amounts of PA (10, 20, and 40 μL) or its fractions (40 μL) were added into 30 mL washed cell suspension. Following the culturation at 25°C for 2 h in the dark, the suspension was analyzed by a fluorescence spectrophotometer (F-4600, Hitachi, Japan) with excitation at 488 nm and emission at 525 nm. Both positive (ROSup, 50 mg/L of final concentration) and negative (Milli-Q water) suspensions were used as the controls in the ROS detection. All treatments were tested in biological triplicates.

Text S5. Microstructure observation of mixed bacteria strains by transmission electron microscope (TEM) and measurement of cell membrane permeability

TEM was used to characterize bacterial morphology in the presence of PA and its fractions.[8] Briefly, a given amount of PA (0, 10, 20, 40 μL) or its fractions (0 and 40 μL) was separately added into 30 mL of the mixed donor and recipient mating culture (1:1, v/v) as described in section 2.3. After 18 h conjugation, the bacterial suspension was washed with sterilized Milli-Q water three times and the concentration was adjusted to 10^6 CFU/mL. The suspension was dripped on a 300-mesh copper coated grid and dried for 5 min,[8] which was then operated by the TEM (HT7700, Hitachi, Japan) at 120 kV to get TEM images.

Cell membrane permeability was determined by a flow cytometry (BD accuri C6, Biosciences, USA) integrating with the propidium iodide dye (PI, Life Technologies, USA).[1] In brief, 1 mL bacterial cell suspensions at 10^6 CFU/mL in PBS solution were obtained after washing the collected cells exposed with 10, 20, and 40 μL PA or its fractions in the above 30-mL mating system for 18 h. Then, 5 μL of 1 mg/mL PI dye was mixed with 1 mL prepared cell solution, and then incubated at 25°C for 15 min in the dark. After staining, the PI fluorescence intensity of bacterial solution was measured by the flow cytometry at 488 nm-excitation and 635 nm-emission. The bacterial cells untreated by PA or its fractions were setup as the control. All samples were conducted in biological triplicates.

Text S6. Measurement of extracellular polymeric substances (EPS)

To obtain the contents of EPS, a heat extraction method was used to extract EPS from the bacteria suspensions.[9] Briefly, 30 mL of the mixed donor and recipient solution under the exposure of 20 and 40 μL PA or its fractions was incubated for 18 h. Then the bacterial suspensions were centrifuged at 4000 rpm and 25°C for 10 min to remove the supernatant, and the bacteria pellets were washed twice with PBS (pH 7.2). Subsequently, the bacterial pellets were resuspended in 5 mL sterile NaCl solution (0.05%) and placed in a 60°C water bath for 30 min. Then the mixture was centrifugated at 4500 rpm for 5 min to collect the supernatant, which was further filtered by a 0.22-μm polyethersulfone filter membrane to remove remaining cells. The filtrate containing EPS was used for further measurement of the contents of proteins and polysaccharides. Considering that EPS has very few components other than proteins and polysaccharides determining bacterial cell aggregation, proteins and polysaccharides were chosen as the representative components of EPS.[10] The content of proteins was detected by Detergent Compatible Bradford Protein Assay Kit (Shanghai Beyotime Biotechnology Co., Ltd., China).[11] Ten μL EPS solution and 300 μL protein working solution were mixed in 96-well plates and the absorbance at 595 nm of mixture was measured by microplate reader (Thermo1500, USA). The polysaccharide contents of extracted EPS solution were analyzed by the phenol-sulfuric acid method.[11] In detail, 1 mL EPS solution was mixed well with 1 mL 5% (v%) phenol in a glass test tube. Then 5 mL 98% sulphuric acid was quickly added to the homogeneous mixture and the tube was shaken well immediately. The tube was then placed in 40°C water bath for 30 min. After that the absorbance at 490 nm of the mixture was detected by a UV-visible spectrophotometer (UV-3300PC, MAPADA, China).

Text S7. Inhibited bacterial activity by PA and the fractions

Higher bacterial concentrations mean more opportunity for the cell-cell contact, which is an important factor on the successful occurrence of conjugative transfer.[12,13] Thus, the viability of *E. coli* strains exposed to

PA and the fractions was examined. From the plate counting, the number of recipient and donor showed amount-dependent reduction by PA and its fractions with an order of PA > F3 > F2 > F1 (Figure 2c, d), consistent with the order effect on conjugative transfer. Typically, the number of recipient and donor decreased by 0.06–0.90 and 0.18–0.59 folds following the high amounts (40–100 μL) of PA and its fractions exposure and by 0.03–0.43 and 0.01–0.41 folds at the low amounts (10–20 μL) of PA and its fractions in the mating system (Figure 1c, d and Figure 2c, d). According to the LCSM images, PA and its fractions also showed that as the increased amounts of PA and its fractions, the red fluorescence area increased gradually (Figure S4). In addition, the red fluorescence area under PA exposure is larger than under its fraction exposure. These results implied that the concentrations of living bacteria reduced gradually with increasing amounts of PA and its fractions, and PA posed greater extent than its fractions in inhibiting bacterial growth. Additionally, the exposure of PA and its fractions decreased the maximum environmental capacity, the maximum specific growth rate and the bacterial concentrations of the donor, recipient and transconjugants in an amount-dependent manner, indicating that bacterial growth was inhibited by PA and its fractions (Figure S5, Table S4). Especially, 40 μL of PA and F3 respectively inhibited the growth of 64.4% and 50.9% of the recipient *E. coli* NK5449, and 56.3% and 42.8% of donor *E. coli* HB101 (Figure S5c, f, i). Moreover, the MIC_{50} of PA to donor, recipient and transconjugants were 2.36, 2.72 and 2.64 μL/mL respectively, and less than its three fractions (Table S1). These results evidenced that the effective inactivation of the recipient and donor bacteria strains by high amount of PA and its fractions, following an order of PA > F3> F2 > F1.

Table S1. Summary of the applied amounts of PA in reported studies.

Feedstock	Application	Applied amounts	Applied results	Reference
Branches of *Xylosma congesta (Lour.) Merr.*	Antimicrobial agents	0.39–12.50 μL/mL	Killed pathogens	[14]
Hybrid aspen clones trees	Herbicide	5%, 12.5%, 25%, and 50% (v/v)	Effective weeding with 25% and 50%	[15]
	Fungicidal agents	5%, 12.5%, 25%, 50% (v/v)	Effective fungicide with 50%	
Blended wood	Agri-chemicals	1% and 2%	Improved the fermentation quality of silages	[16]
Blended wood wastes	Soil amendments	7.17–74.2%	Reduced ARG level, weakened horizontal gene transfer, decreased co-selection of heavy metals and shifted bacterial community	[17]
Blended wood wastes	Soil amendments	0.2%	Improved the nutritional quality and the soil nutrient availability	[18]

Table S2. Minimum inhibitory concentrations (MICs) of PA, the three distilled fractions, and the four representative components.

Bacteria strians	MICs	PA[β] (μL/mL)	F1 (μL/mL)	F2 (μL/mL)	F3 (μL/mL)	Acetic acid[γ] (mg/mL)	2-methoxy-phenolx (mg/mL)	2, 6-dimethoxy phenol (mg/mL)	3-methyl-1, 2-cyclopentanedione (mg/mL)
E. coli NK5449	MIC_{10}[α]	0.706	1.65	1.05	1.35	0.004	0.037	0.270	0.484
	MIC_{50}	2.78	4.50	3.46	3.32	0.079	0.446	0.768	1.89
	MIC_{90}	4.85	7.35	5.86	5.28	0.153	0.929	1.41	3.29
E. coli HB101	MIC_{10}	0.807	1.68	1.33	1.21	0.002	0.004	0.170	0.059
	MIC_{50}	2.40	3.92	3.19	2.79	0.074	0.457	0.678	1.11
	MIC_{90}	4.00	6.16	5.06	4.36	0.145	0.911	1.52	2.16

[α] $MIC_{10/50/90}$: the 10%, 50%, and 90% minimum inhibitory concentration of PA, the three distilled fractions, and their four representative components to recipient *E. coli* NK5449 or donor *E. coli* HB101.

[β] PA: pyroligneous acid derived from the blended woody waste collected from furniture factory at 450°C for 6 h; F1, F2, and F3: the fraction of PA collected using atmospheric distillation at 98, 130, and 220°C, respectively.

[γ] Acetic acid (> 99.5% pure), guaiacol (99% pure), 2,6-dimethoxyphenol (98% pure), and 3-methyl-1,2-cyclopentadione (98% pure): four representative components of the PA and the distilled fractions, which were purchased from Sinopharm Chemical Reagent Co., Ltd, China, and Guangzhou Chemical Reagent Factory, China.

Table S3. The transconjugants number, conjugative transfer frequency, recipient and donor number in a 30-mL mating system under exposure of PA.

PA[α] amounts (μL)	Transconjugants number (CFU/mL)	Conjugative transfer frequency[β]	Recipient *E. coli* NK5449 number (CFU/mL)	Donor *E. coli* HB101number (CFU/mL)
0	3.53×10^{5}	6.79×10^{-5}	5.21×10^{9}	1.12×10^{8}
10	$4.43 \times 10^{5\,*\gamma}$	$1.45 \times 10^{-4\,**}$	$3.07 \times 10^{9\,**}$	$1.02 \times 10^{8\,**}$
20	$5.19 \times 10^{5\,**}$	$2.27 \times 10^{-4\,**}$	$2.30 \times 10^{9\,**}$	$8.96 \times 10^{7\,**}$
40	$9.14 \times 10^{4\,**}$	6.94×10^{-5}	$1.32 \times 10^{9\,**}$	$8.68 \times 10^{7\,**}$
60	$6.97 \times 10^{4\,**}$	$9.17 \times 10^{-5\,*}$	$7.50 \times 10^{9\,**}$	$5.96 \times 10^{7\,*}$
80	$5.24 \times 10^{4\,**}$	8.80×10^{-5}	$8.49 \times 10^{9\,**}$	$6.71 \times 10^{7\,**}$
100	$5.48 \times 10^{3\,**}$	$9.85 \times 10^{-5\,*}$	$5.32 \times 10^{9\,**}$	$4.59 \times 10^{7\,**}$

[α] PA: the pyroligneous acid prepared from pyrolysis of blended woody waste collected from furniture factories at 450°C for 6 h.

[β] Conjugative transfer frequency: the conjugative transfer frequency of plasmid RP4 between the donor *E. coli* HB101 and recipient *E. coli* NK5449 in a 30-mL mating system was calculated as the ratio of the transconjugant number to the total recipient number. The mating conditions of conjugation: 10^{8} CFU/mL *E. coli* HB101 as donor and 10^{8} CFU/mL *E. coli* NK5449 as recipient, which were mixed at a volume ratio of 1:1 and incubated at 37°C for 18 h.

[γ] The asterisks indicated significant differences between the PA treatments with control group (independent sample *t*-test, n =3, * for $P < 0.05$, ** for $P < 0.01$).

Table S4. The transconjugants number, conjugative transfer frequency, recipient and donor number in a 30-mL mating system under exposure of PA and its three fractions.

Amounts (μL)	Samples	Transconjugants number (CFU/mL)	Conjugative transfer frequency[β]	Recipient *E. coli* NK5449 number (CFU/mL)	Donor *E. coli* HB101number (CFU/mL)
10	CK	4.64×10^{5}	9.60×10^{-5}	4.84×10^{9}	1.00×10^{8}
	PA[α]	6.93×10^{5} **[γ]	2.51×10^{-4} **	2.78×10^{9} **	5.79×10^{7} **
	F1	5.87×10^{5} **	1.25×10^{-4} **	4.70×10^{9}	9.87×10^{7}
	F2	6.10×10^{5} **	1.40×10^{-4} **	4.37×10^{9} *	7.67×10^{7}
	F3	5.82×10^{5} **	1.48×10^{-4} **	3.93×10^{9} **	7.97×10^{7}
20	CK	3.44×10^{5}	1.71×10^{-4}	2.01×10^{9}	1.03×10^{8}
	PA	6.31×10^{5} **	5.10×10^{-4} **	1.24×10^{9} **	7.91×10^{7} **
	F1	3.27×10^{5}	1.72×10^{-4}	1.90×10^{9} *	9.87×10^{7}
	F2	5.04×10^{5} **	2.98×10^{-4} **	1.69×10^{9} **	9.32×10^{7}
	F3	5.59×10^{5} **	3.82×10^{-4} **	1.46×10^{9} **	8.63×10^{7} **
40	CK	3.43×10^{5}	1.80×10^{-4}	1.92×10^{9}	9.35×10^{7}
	PA	9.41×10^{4} **	1.38×10^{-4}	6.83×10^{8} **	4.08×10^{7} **
	F1	2.08×10^{5} **	1.16×10^{-4} *	1.79×10^{9}	7.67×10^{7} **
	F2	1.91×10^{5} **	1.31×10^{-4}	1.46×10^{9} **	6.46×10^{7} **
	F3	1.74×10^{5} **	1.86×10^{-4}	9.42×10^{8} **	5.35×10^{7} **

[α] PA: the pyroligneous acid prepared from pyrolysis of blended woody waste collected from furniture factories at 450°C for 6 h; F1, F2, and F3: the fraction of PA collected using atmospheric distillation at 98, 130, and 220°C, respectively.

[β] Conjugative transfer frequency: the conjugative transfer frequency of plasmid RP4 between the donor *E. coli* HB101 and recipient *E. coli* NK5449 in a 30-mL mating system was calculated as the ratio of the transconjugant number to the total recipient number. The mating conditions of conjugation: 10^{8} CFU/mL *E. coli* HB101 as donor and 10^{8} CFU/mL *E. coli* NK5449 as recipient, which were mixed at a volume ratio of 1:1 and incubated at 37°C for 18 h.

[γ] The asterisks indicated significant differences between the PA and distilled fraction treatments with control group (independent sample *t*-test, n =3, * for $P < 0.05$, ** for $P < 0.01$).

Table S5. The transconjugants number, conjugative transfer frequency, recipient and donor number in a 30-mL mating system under exposure of four representative components.

Representative components	Concentrations (mg/mL)	Transconjugants number (CFU/mL)	Conjugative transfer frequency[β]	Recipient *E. coli* NK5449 number (CFU/mL)	Donor *E. coli* HB101number (CFU/mL)
Acetic acid[α]	0	1.61×10^{6}	1.45×10^{-3}	1.13×10^{9}	3.54×10^{8}
	0.001	2.25×10^{6}	1.86×10^{-3}	1.24×10^{9}	2.90×10^{8}
	0.01	1.08×10^{6}	1.46×10^{-3}	7.50×10^{8}	2.04×10^{8}
	0.1	2.32×10^{2} **[γ]	3.94×10^{-5} **	5.90×10^{6} *	2.00×10^{6} *
Guaiacol	0	2.74×10^{5}	1.30×10^{-4}	2.15×10^{9}	4.27×10^{8}
	0.01	4.10×10^{5} *	2.54×10^{-4} **	1.63×10^{9}	4.26×10^{8}
	0.1	4.72×10^{5} **	3.17×10^{-4} **	1.50×10^{9} *	2.56×10^{8} **
	0.5	3.73×10^{5}	3.15×10^{-4} *	1.19×10^{9} *	1.76×10^{8} **
	1	6.74×10^{4} **	1.24×10^{-4}	5.43×10^{8} **	7.11×10^{7} **
2,6-dimethoxyphenol	0	1.30×10^{6}	6.84×10^{-4}	1.90×10^{9}	5.93×10^{8}
	0.1	1.45×10^{6}	1.05×10^{-3} *	1.40×10^{9}	4.44×10^{8} *
	0.5	1.14×10^{6}	1.37×10^{-3} **	8.32×10^{8} *	3.19×10^{8} **
	1	6.27×10^{5} *	2.87×10^{-3} **	2.19×10^{8} *	1.10×10^{8} **
	2	1.18×10^{3} *	7.83×10^{-5} **	1.51×10^{7} *	1.25×10^{7} **
3-methyl-1,2-cyclopentadione	0	4.69×10^{5}	2.30×10^{-4}	2.05×10^{9}	4.82×10^{8}
	0.05	5.79×10^{5} *	2.94×10^{-4}	2.02×10^{9}	4.50×10^{8}
	0.5	5.84×10^{5} *	3.30×10^{-4} *	1.78×10^{9}	2.69×10^{8} **
	1.5	2.29×10^{5} **	1.66×10^{-4}	1.39×10^{9} **	1.39×10^{8} **
	3	7.19×10^{4} **	7.50×10^{-5} *	9.59×10^{8} **	5.11×10^{6} **

[α] Acetic acid (> 99.5% pure), guaiacol (99% pure), 2,6-dimethoxyphenol (98% pure), and 3-methyl-1,2-cyclopentadione (98% pure): four representative components of the PA and the distilled fractions, which were purchased from Sinopharm Chemical Reagent Co., Ltd, China, and Guangzhou Chemical Reagent Factory, China.

[β] Conjugative transfer frequency: the conjugative transfer frequency of plasmid RP4 between the donor *E. coli* HB101 and recipient *E. coli* NK5449 in a 30-mL mating system was calculated as the ratio of the transconjugant number to the total recipient number. The mating conditions of conjugation: 10^{8} CFU/mL *E. coli* HB101 as donor and 10^{8} CFU/mL *E. coli* NK5449 as recipient, which were mixed at a volume ratio of 1:1 and incubated at 37°C for 18 h.

[γ] The asterisks indicated significant differences between the four representative components treatments with control group (independent sample *t*-test, n =3, * for $P < 0.05$, ** for $P < 0.01$).

Table S6. The pHs of three mating systems with different treatments.

Samples	Amounts (μL)	Unadjusted-pH group[α]	Adjusted-pH group[β]	No-PA group[γ]
PA[δ]	0	7.00	7.00	7.00
	20	6.34	7.00	6.34
	40	6.14	7.00	6.14
F1	0	7.00	7.00	7.00
	20	6.75	7.00	6.75
	40	6.45	7.00	6.45
F2	0	7.00	7.00	7.00
	20	6.54	7.00	6.54
	40	6.21	7.00	6.21
F3	0	7.00	7.00	7.00
	20	6.07	7.00	6.07
	40	5.45	7.00	5.45

[α] Unadjusted-pH group: the treatments added with PA or its fractions (20 and 40 μL) without any pH adjustment.
[β] Adjusted-pH group: the treatments added with PA or its fractions, and the pHs were adjusted to 7.0 as the control group by 0.1 M NaOH.
[γ] No-PA group: the treatments without PA or its fraction addition, and the pHs were adjusted as same as those containing corresponding PA or its fractions by 0.1 M HCl.
[δ] PA: pyroligneous acid derived from the blended woody waste collected from furniture factory at 450°C for 6 h; F1, F2, and F3: the fraction of PA collected using atmospheric distillation at 98, 130, and 220°C, respectively.

Table S7. The pHs of four representative components with different concentrations in Milli-Q water.

Samples	Concentrations (mg/mL)	pH
acetic acid[α]	0.001	4.53
	0.010	4.09
	0.100	3.54
2-methoxy-phenol	0.010	6.85
	0.100	6.62
	0.500	6.48
	1.000	6.34
2, 6-dimethoxy phenol	0.100	6.63
	0.500	6.40
	1.000	6.35
	2.000	6.22
3-methyl-1, 2-cyclopentanedione	0.050	6.80
	0.500	6.41
	1.500	6.17
	3.000	6.01

[α] Acetic acid (> 99.5% pure), guaiacol (99% pure), 2,6-dimethoxyphenol (98% pure), and 3-methyl-1,2-cyclopentadione (98% pure): four representative components of the PA and the distilled fractions, which were purchased from Sinopharm Chemical Reagent Co., Ltd, China, and Guangzhou Chemical Reagent Factory, China.

Table S8. Fitting parameters of growth curves of *E. coli* under PA and its fractions by logistic growth mode.

	Amounts[α] (μL)	Bacteria	Slogistic $y = a/(1 + \exp(-k*(x-x_c)))$				
			a^{β}	k^{γ}	x_c^{δ}	$\vartheta_{max}^{\varepsilon}$	R^2
PA[ζ]	0	*E. coli* HB101	1.28	0.504	4.78	0.161	0.980
		E. coli NK5449	1.57	0.421	5.45	0.165	0.965
		Transconjugation	1.89	0.856	4.33	0.404	0.974
	10	*E. coli* HB101	1.20	0.324	5.92	0.097	0.953
		E. coli NK5449	1.31	0.436	5.07	0.143	0.953
		Transconjugation	1.66	0.700	4.63	0.290	0.967
	20	*E. coli* HB101	1.11	0.338	5.79	0.094	0.952
		E. coli NK5449	1.29	0.421	5.23	0.136	0.952
		Transconjugation	1.53	0.776	4.49	0.297	0.973
	40	*E. coli* HB101	1.03	0.349	5.69	0.090	0.943
		E. coli NK5449	1.22	0.415	5.33	0.127	0.947
		Transconjugation	1.49	0.666	4.88	0.248	0.972
F1	0	*E. coli* HB101	1.28	0.504	4.78	0.161	0.980
		E. coli NK5449	1.57	0.421	5.45	0.165	0.965
		Transconjugation	1.89	0.856	4.33	0.404	0.974
	10	*E. coli* HB101	1.24	0.486	4.79	0.151	0.975
		E. coli NK5449	1.41	0.447	5.05	0.158	0.953
		Transconjugation	1.86	0.773	4.50	0.359	0.974
	20	*E. coli* HB101	1.22	0.424	5.10	0.129	0.975
		E. coli NK5449	1.49	0.428	5.63	0.159	0.975
		Transconjugation	1.78	0.841	4.39	0.374	0.974
	40	*E. coli* HB101	1.20	0.404	5.25	0.121	0.972
		E. coli NK5449	1.50	0.424	5.73	0.159	0.975
		Transconjugation	1.72	0.864	4.50	0.371	0.970
F2	0	*E. coli* HB101	1.28	0.504	4.78	0.161	0.980
		E. coli NK5449	1.57	0.421	5.45	0.165	0.965
		Transconjugation	1.89	0.856	4.33	0.404	0.974
	10	*E. coli* HB101	1.22	0.389	5.34	0.119	0.966
		E. coli NK5449	1.38	0.424	5.02	0.146	0.961
		Transconjugation	1.79	0.722	4.63	0.323	0.975
	20	*E. coli* HB101	1.19	0.386	5.26	0.115	0.960
		E. coli NK5449	1.36	0.427	5.05	0.145	0.962
		Transconjugation	1.71	0.734	4.54	0.314	0.970

	40	*E. coli* HB101	1.16	0.355	5.54	0.103	0.956
		E. coli NK5449	1.33	0.454	5.01	0.151	0.957
		Transconjugation	1.61	0.861	4.45	0.346	0.980
F3	0	*E. coli* HB101	1.28	0.504	4.78	0.161	0.980
		E. coli NK5449	1.57	0.421	5.45	0.165	0.965
		Transconjugation	1.89	0.856	4.33	0.404	0.974
	10	*E. coli* HB101	1.21	0.356	5.56	0.108	0.956
		E. coli NK5449	1.35	0.440	5.10	0.149	0.960
		Transconjugation	1.71	0.764	4.46	0.327	0.977
	20	*E. coli* HB101	1.08	0.422	4.96	0.114	0.961
		E. coli NK5449	1.32	0.441	5.19	0.146	0.961
		Transconjugation	1.64	0.801	4.65	0.328	0.982
	40	*E. coli* HB101	1.06	0.388	5.38	0.103	0.961
		E. coli NK5449	1.23	0.468	4.96	0.135	0.951
		Transconjugation	1.57	0.726	5.18	0.285	0.981

[α] Amounts: 150 μL of donor and recipient bacteria suspensions were respectively added into 30 mL LB liquid medium containing different amounts (10, 20, and 40 μL) of PA or its three distilled fractions.

[β] a: maximum environmental capacity.

[γ] k: growth rate which the y closes to maximum environmental capacity.

[δ] x_c: the time at which the growth rate is greatest.

[ε] ϑ_{max}: the maximum specific growth rate.

[ζ] PA: pyroligneous acid derived from the blended woody waste collected from furniture factory at 450°C for 6 h; F1, F2, and F3: the fraction of PA collected using atmospheric distillation at 98, 130, and 220°C, respectively.

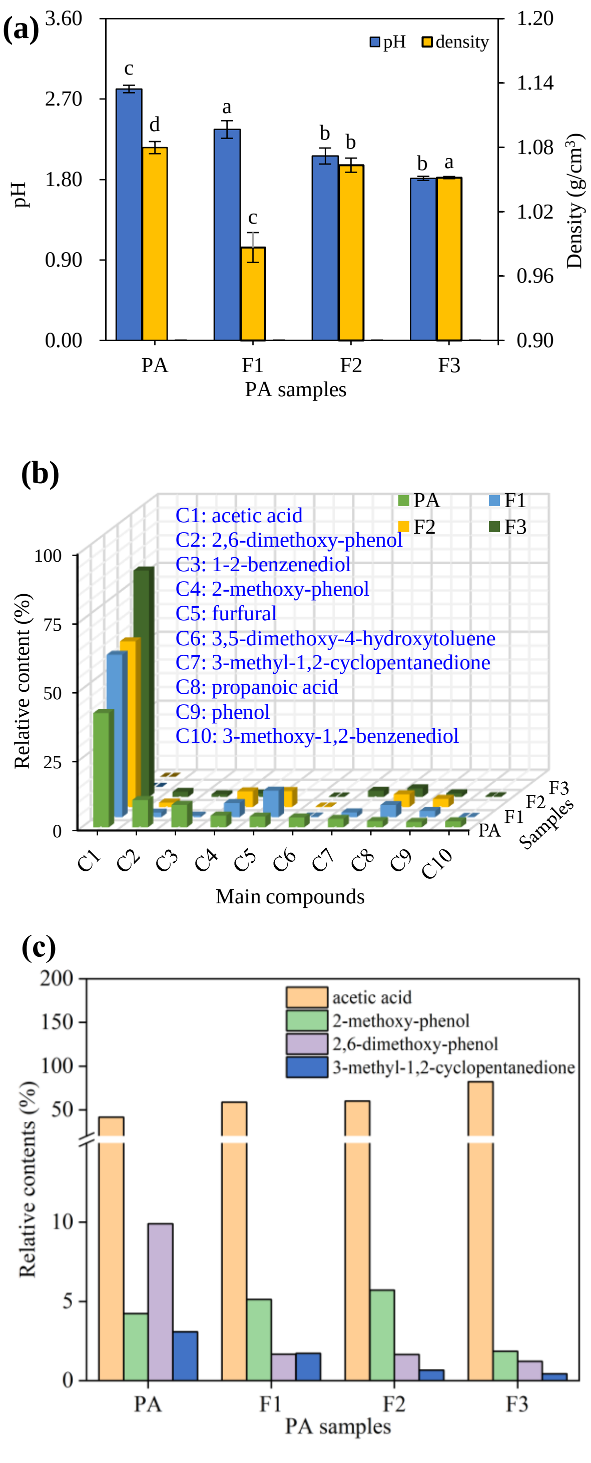

Figure S1. Properties of PA and its distilled fractions as previous reported.[17] (a) Density and pH of PA and its distilled fractions; (b) the top ten compounds in the PA samples; (c) the relative contents of four representative components (i.e., acetic acid, 2-methoxy-phenolx, 2,6-dimethoxy-phenol, and 3-methyl-1,2-cyclopentanedione) of PA. PA: pyroligneous acid from pyrolyzing blended woody waste at 450 °C for 6 h; F1, F2, and F3: distilled fractions of PA at 98, 130, and 220 °C, respectively. The different small letters represented significant difference among the different treatments (Duncan's multiple-comparison test, $n = 3$, $P < 0.05$).

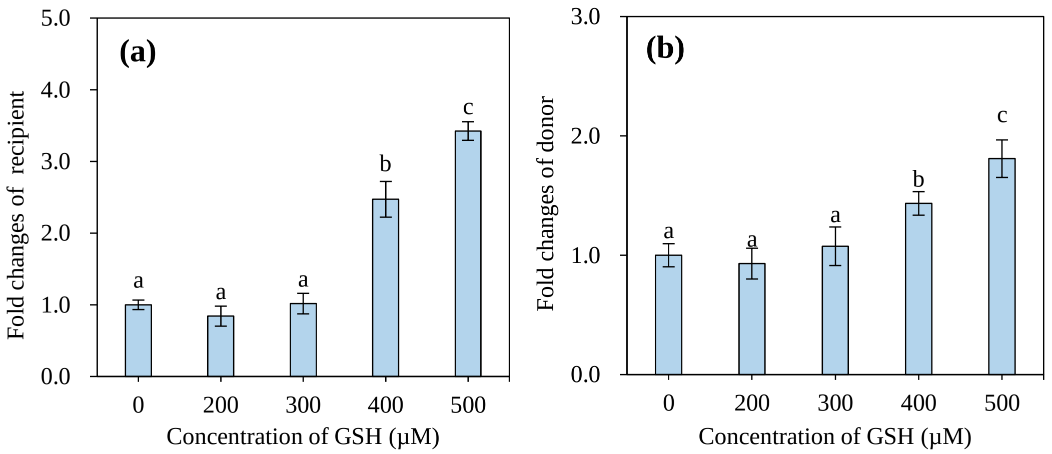

Figure S2. Effect of ROS scavenger glutathione (GSH) at different concentrations on the growth of (a) recipient *E. coli* NK5449 and (b) donor *E. coli* HB101. The culture conditions: 10^8 CFU/mL *E. coli* HB101 as donor and 10^8 CFU/mL *E. coli* NK5449 as recipient, for 18 h at 37°C. The different small letters represented significant difference among the different treatments (Duncan's multiple-comparison test, $n = 3$, $P < 0.05$).

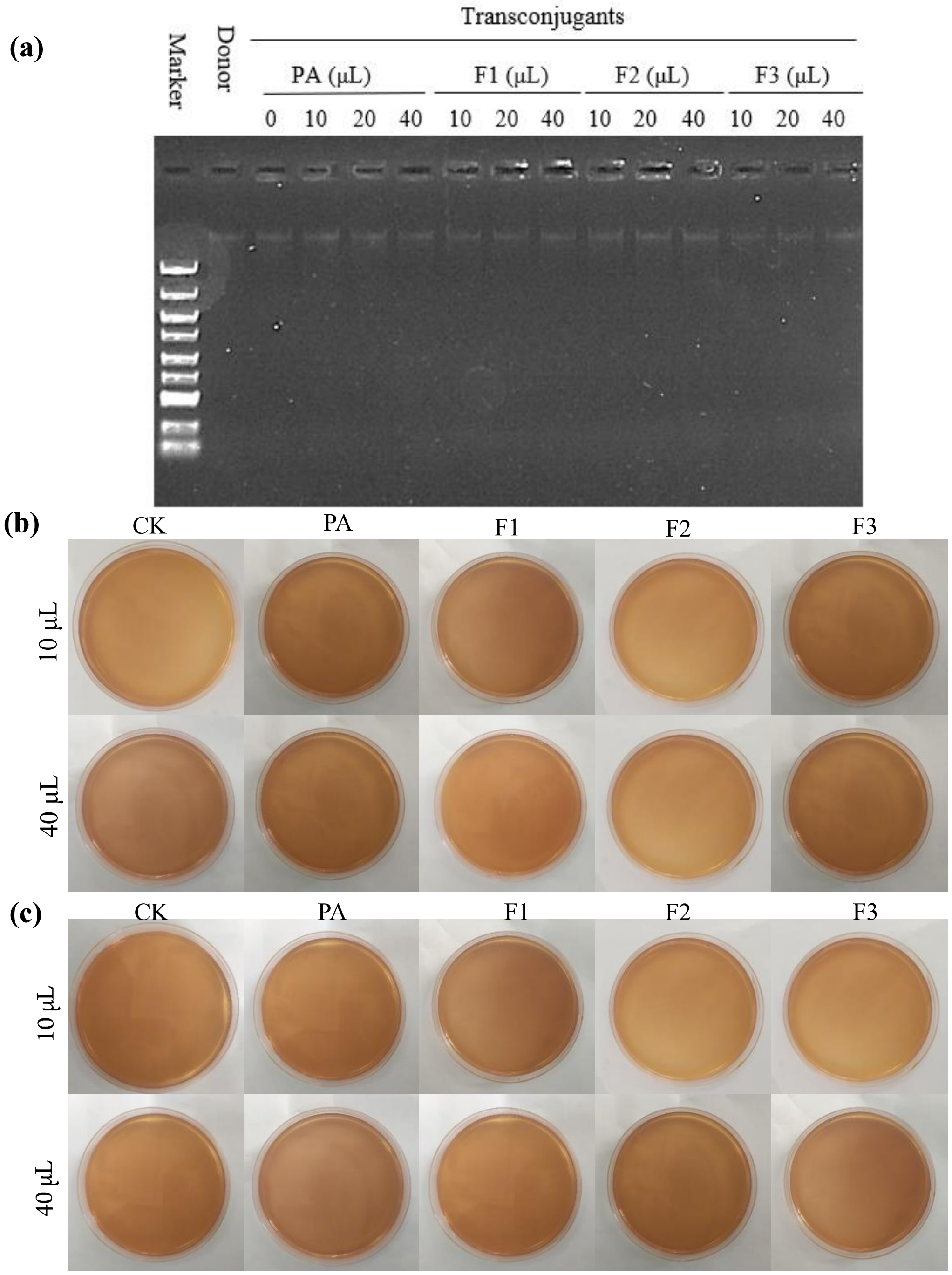

Figure S3. Evidence of transconjugants production under PA and its fractions exposure. Gel electrophoresis photos (a) of the RP4 plasmids extracted from the donor *E. coli* HB101 and transconjugants. The photos of transconjugant selection LB-agar plates with the donor *E. coli* HB101 (b) and recipient *E. coli* NK5449 (c) spontaneous mutation or mutation induced by PA and its fractions. Clear bands of plasmids RP4 in the transconjugants with the same size as those in the donor bacterial strains were observed, and no mutated donor or recipient strains were observed on the transconjugant selection LB-agar plates, confirming that the transconjugants production under PA and its fractions exposure. The mating conditions: 10^8 CFU/mL *E. coli* HB101 as donor and 10^8 CFU/mL *E. coli* NK5449 as recipient mixed at a ratio of 1:1 (v/v), 37°C for 18 h.

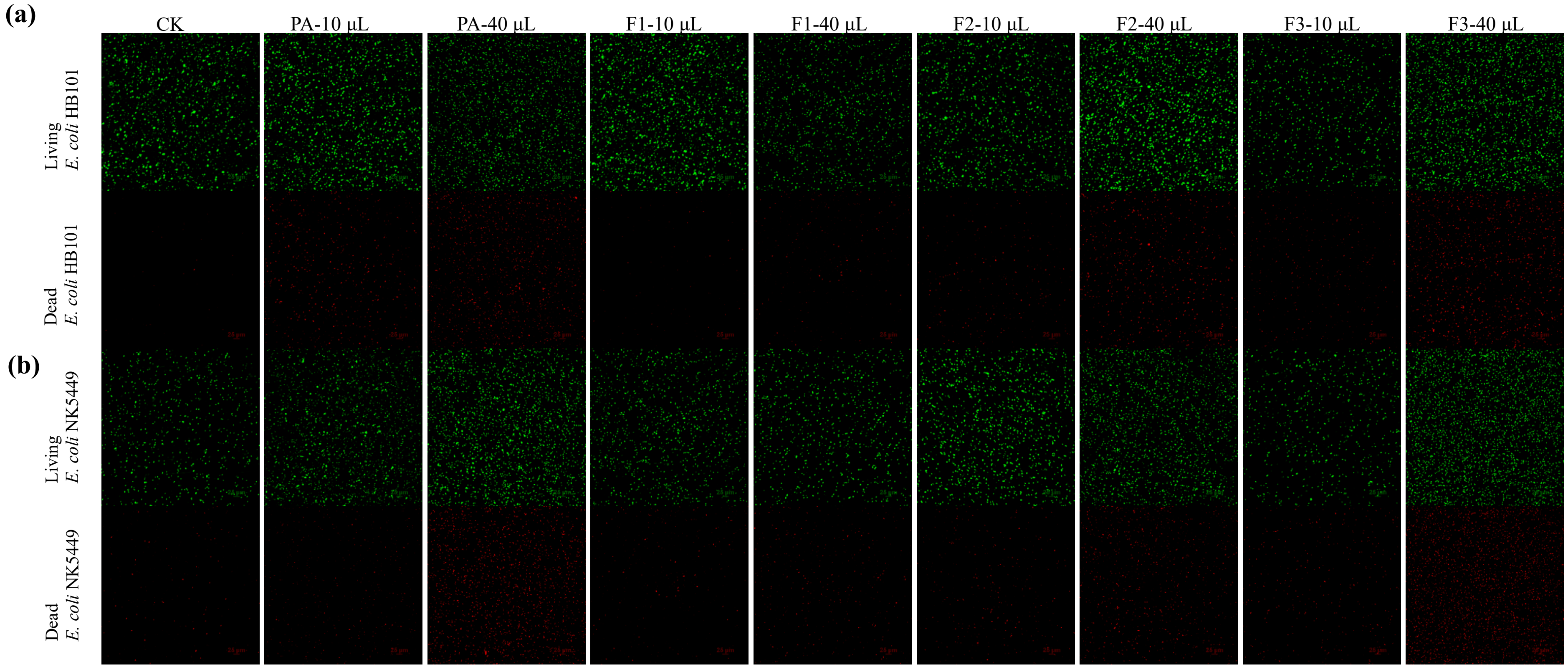

Figure S4. LCSM images of donor *E. coli* HB101 (a) and recipient *E. coli* NK5449 (b) exposed with 0, 10 and 40 μL PA or its fractions in a 30-mL mating system. The first and second row respectively showed the living (green fluorescence) and dead (red fluorescence) donor *E. coli* HB101. The third and fourth row respectively showed the living (green fluorescence) and dead (red fluorescence) recipient *E. coli* NK5449 strains. PA: pyroligneous acid derived from blended woody waste collected from furniture factory at 450°C for 6 h; F1, F2, and F3: the fraction of PA collected using atmospheric distillation at 98, 130, and 220°C, respectively. The bacterial cells were dyed with SYTO 9 and propidium iodide (PI). The stained green and red colours represented the living and dead cells, respectively. The strains were adjusted to approximately 10^8 CFU/mL using LB media and incubated at 37°C for 2 h.

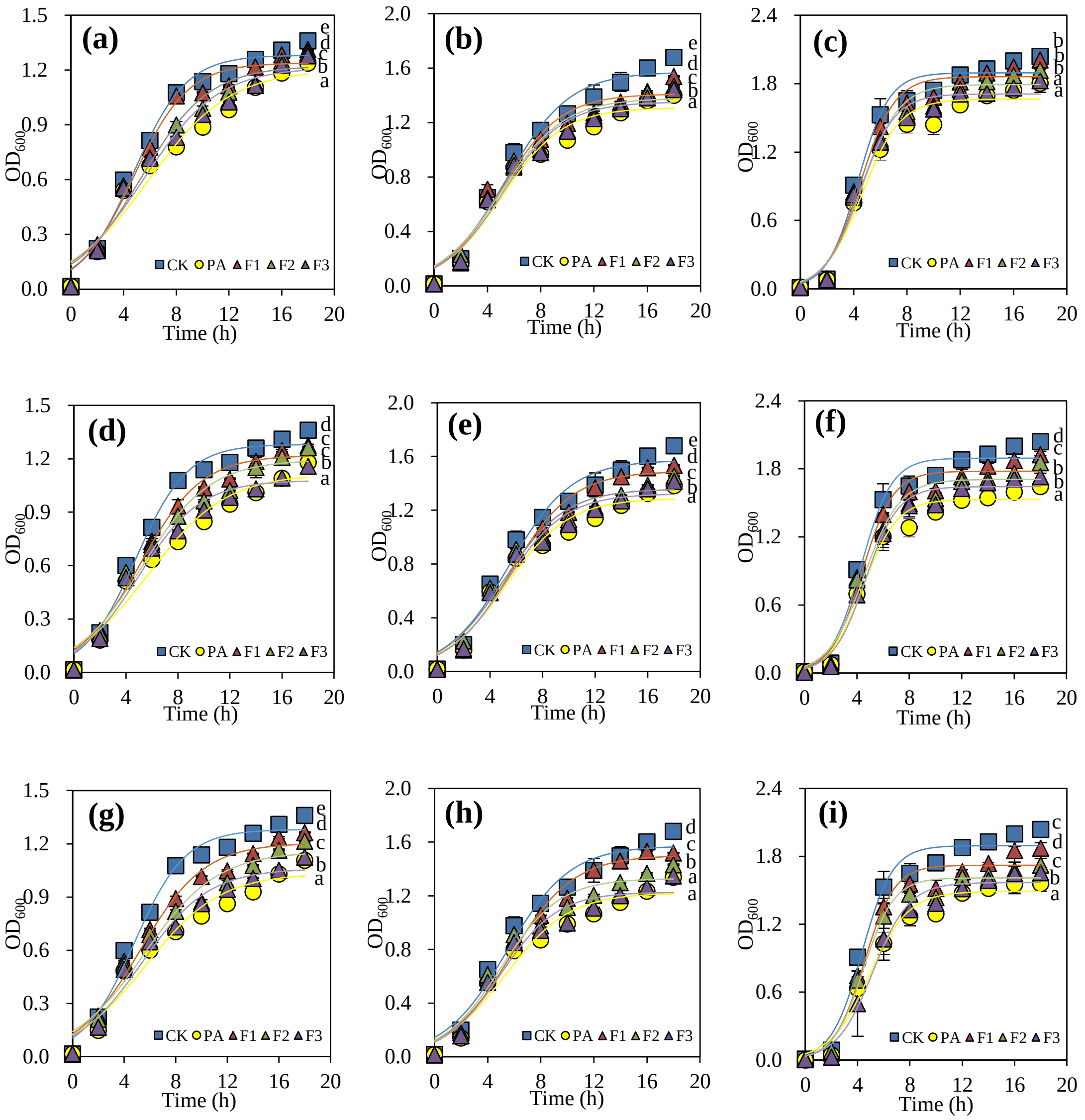

Figure S5. Effects of PA and its fractions on the growth of *E. coli* bacterial strains. Growth curves of (a, b, c) donor, (d, e, f) recipient, and (g, h, i) transconjugant under PA and its fractions exposure at amounts of 10, 20, and 40 μL in a 30-mL mating system were fitted using Logistic growth model. PA: pyroligneous acid derived from blended woody waste collected from furniture factory at 450°C for 6 h; F1, F2, and F3: the fraction of PA collected using atmospheric distillation at 98, 130, and 220°C, respectively. The strains were adjusted to approximately 10^8 CFU/mL using LB media and incubated at 37°C for 18 h. The bacteria growth curve fitted using logistic growth model with Origin 2022. The different small letters represented significant difference among the different treatments (Duncan's multiple-comparison test, $n = 3$, $P < 0.05$).

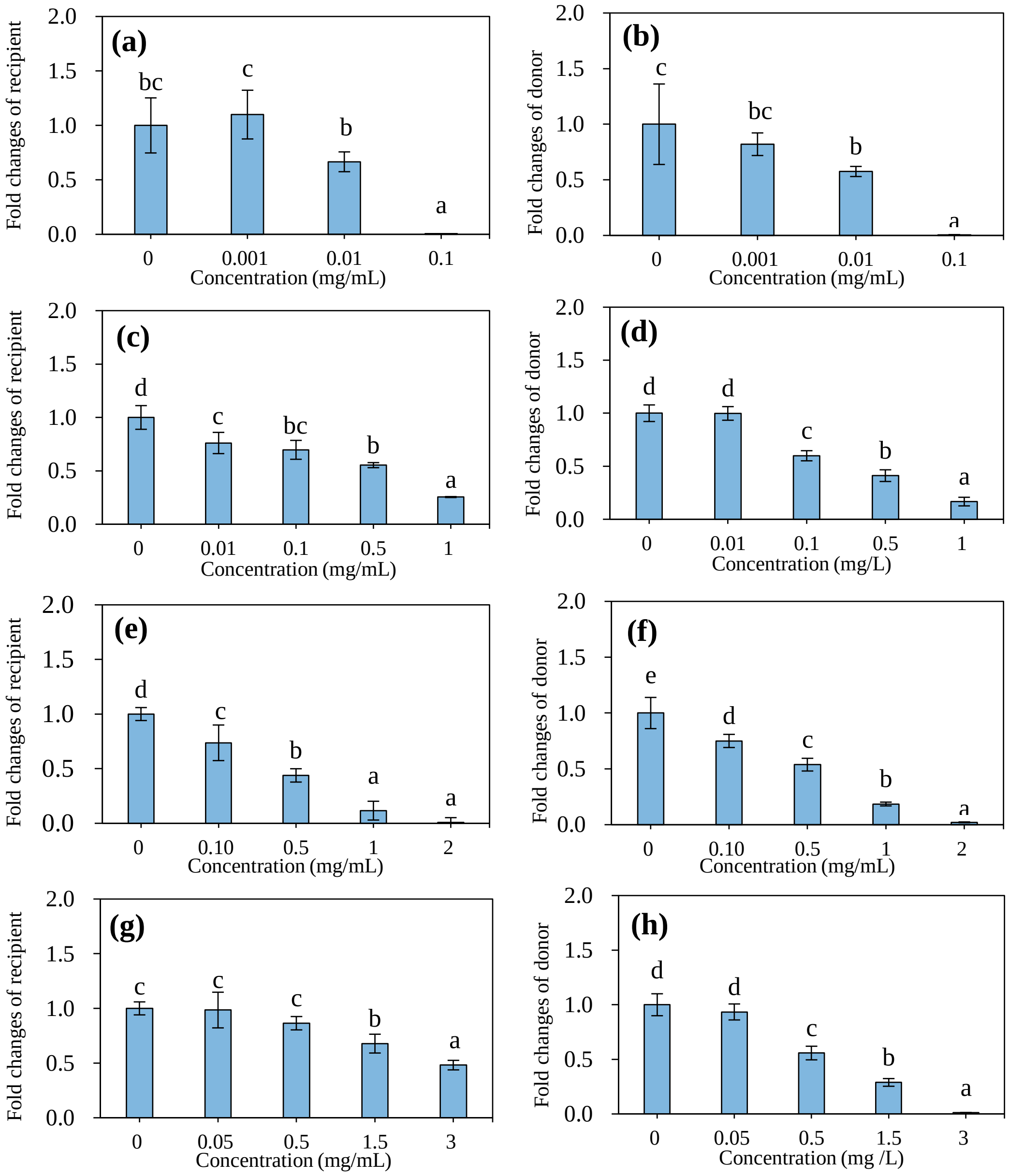

Figure S6. Effects of four representative components of PA on the strain number of the recipient *E. coli* NK5449 and donor *E. coli* HB101. Cytotoxicity of acetic acid (a, b), 2-methoxy-phenolx (c, d), 2,6-dimethoxy-phenol (e, f), and 3-methyl-1,2-cyclopentanedione (g, h) on recipient *E. coli* NK5449 and donor *E. coli* HB101. The mating conditions: 10^8 CFU/mL *E. coli* HB101 as donor and 10^8 CFU/mL *E. coli* NK5449 as recipient mixed at a ratio of 1:1 (v/v), 37°C for 18 h. The different small letters represented significant difference among the treatments exposed with PA and its fraction at the same concentration (Duncan's multiple-comparison test, $n = 3$, $P < 0.05$).

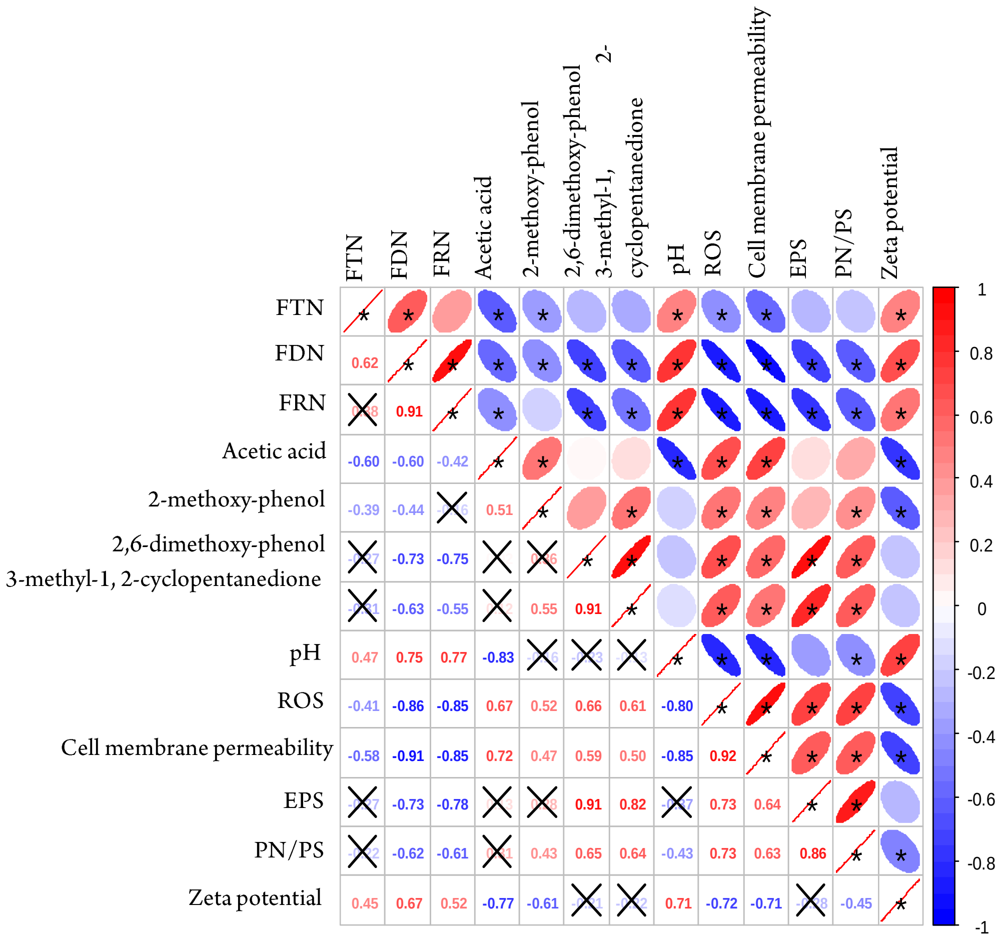

Figure S7. Pearson correlation analysis for exploring the relationships between fold changes of transconjugant number (FTN), fold changes of donor number (FDN), fold changes of recipient number (FRN), acetic acid, 2-methoxy-phenolx, 2, 6-dimethoxy phenol, and 3-methyl-1, 2-cyclopentanedione contents of PA, pHs of mating systems, ROS, cell membrane permeability, EPS, PN/PS, and zeta potential levels of recipient and donor strains. Colors from blue to red represented changes in the Pearson correlation coefficient from −1 to 1. The asterisks indicated significant correlations at $P < 0.05$, and × indicates no significant correlation.

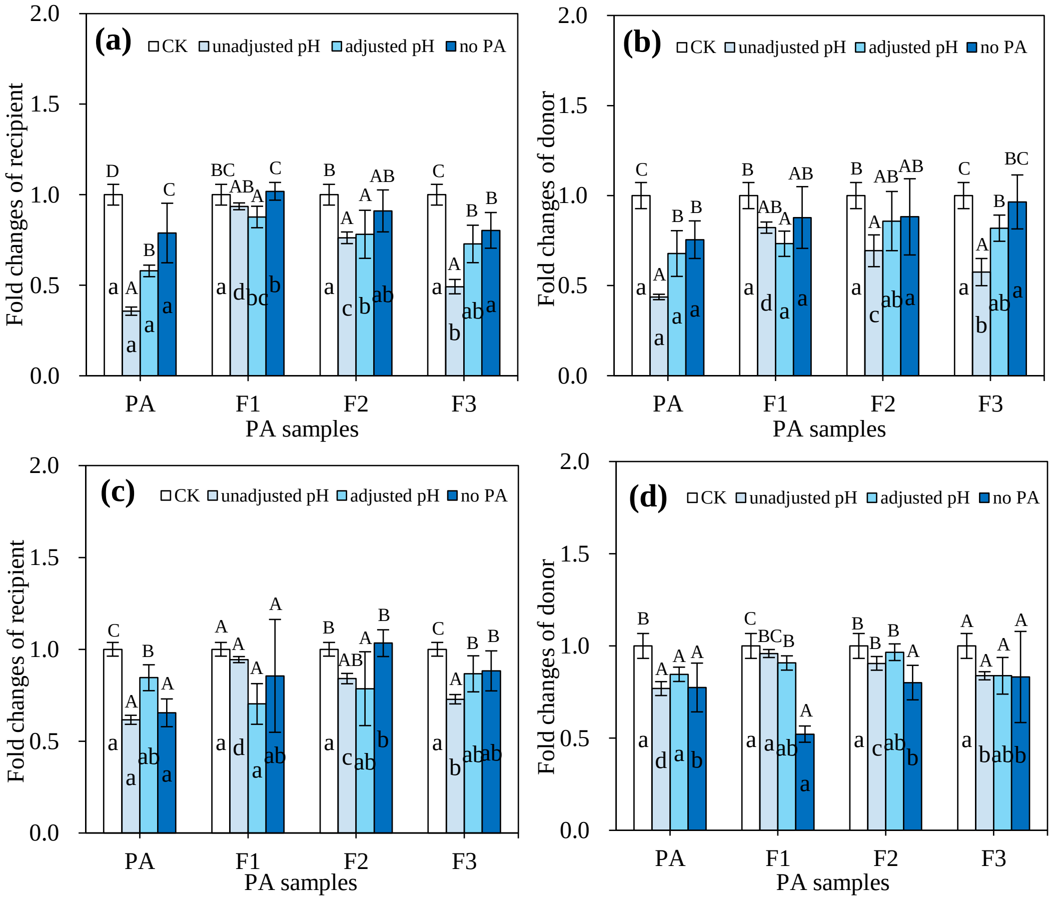

Figure S8. Effects of pH in a 30-mL mating system exposed with PA and its distilled fractions at 40 μL (a, b) and 20 μL (c, d) on the growth of recipient NK5449 and donor *E. coli* HB101. Unadjusted-pH group: the treatments added with PA or its fractions (20, and 40 μL) without any pH adjustment; adjusted-pH group: the treatments added with PA or its fractions, of which pHs were adjusted to 7.0 as the control group by 0.1 M NaOH; no-PA group: the treatments without PA or its fraction addition, of which pHs were adjusted as same as those containing corresponding PA or its fractions by 0.1 M HCl. The mating conditions: 10^8 CFU/mL *E. coli* HB101 as donor and 10^8 CFU/mL *E. coli* NK5449 as recipient mixed at a ratio of 1:1 (v/v), 37°C for 18 h. The different small letters represented significant difference among the different treatments exposed with PA and its fractions, and the capital letters indicated significant difference among the treatments with and without pH adjustment (Duncan's multiple-comparison test, $n = 3$, $P < 0.05$).

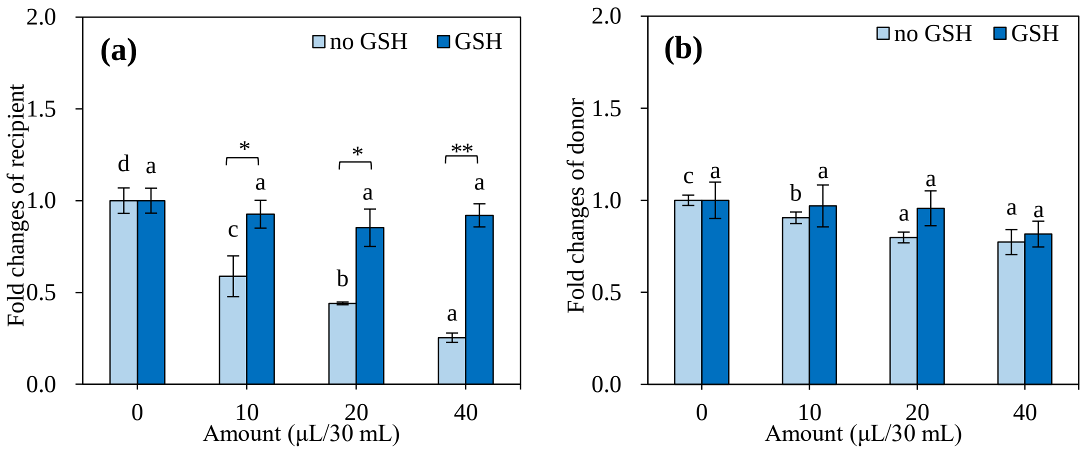

Figure S9. Effect of ROS scavenger glutathione (GSH) on the number of (a) recipient *E. coli* NK5449 and (b) donor *E. coli* HB101 under PA exposure. PA: the pyroligneous acid derived from blended woody waste collected from furniture factory at 450°C for 6 h. The culture conditions: 10^8 CFU/mL *E. coli* HB101 as donor and 10^8 CFU/mL *E. coli* NK5449 as recipient, which were mixed at a volume ratio of 1:1 and incubated at 37°C for 18 h. The different small letters represented significant difference among the different treatments added with PA or the fractions at different amounts (Duncan's multiple-comparison test, $n = 3$, $P < 0.05$), and the asterisks indicated significant differences between the treatments with and without GSH addtion (independent sample *t*-test, $n = 3$, * for $P < 0.05$, ** for $P < 0.01$).

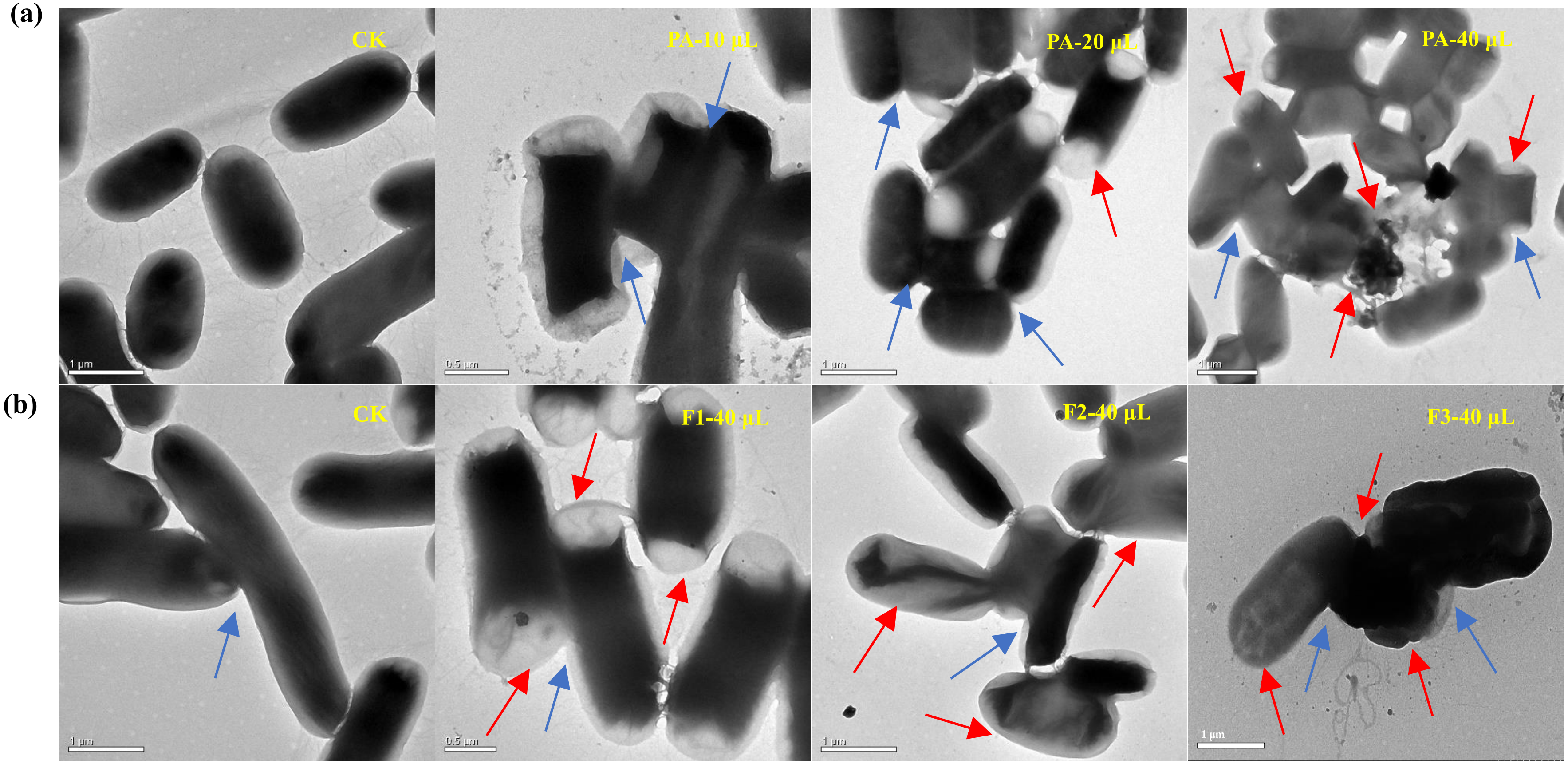

Figure S10. TEM images of the mixed donor *E. coli* HB101 and recipient *E. coli* NK5449 exposed to (a) PA and (b) its distilled fractions. The red arrows indicated the damages of cell membranes, and the blue arrows showed the contact between the bacterial cells. When exposure of low amount (10 and 20 μL) of PA and the three fractions in a 30-mL mating system**,** the cells were obviously observed with damaged cell membranes, emerged pores, unclear cell boundaries and enhanced cell-to-cell contact. At the high amount (40 μL) of PA and the three fractions in the 30-mL mating system, more severely damaged cell membrane, fractured pilus, and shrunken or leaky cytoplasm were observed.

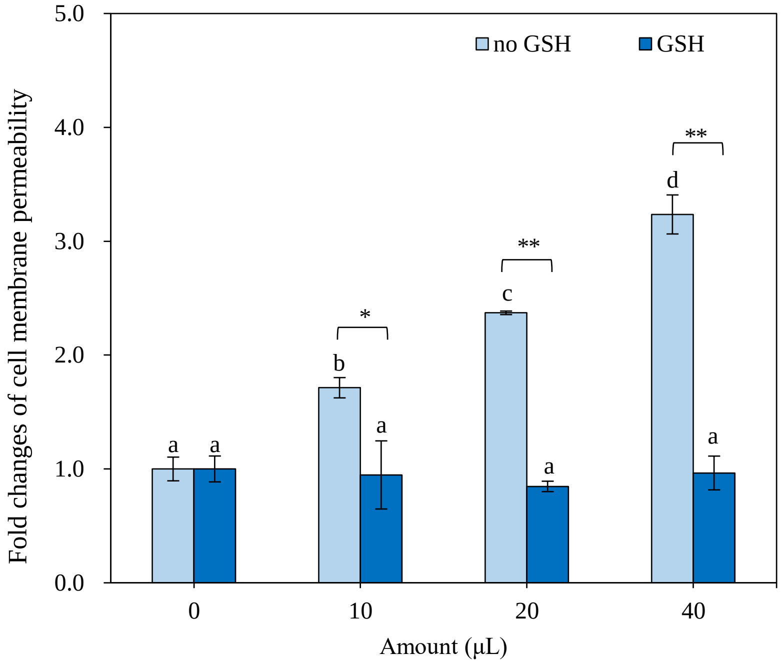

Figure S11. Fold changes in cell membrane permeability of the mixed donor *E. coli* HB101 and recipient *E. coli* NK5449 treated with ROS scavenger GSH (300 μmol/L) under PA exposure. PA: the pyroligneous acid derived from blended woody waste collected from furniture factory at 450°C for 6 h. The culture conditions: 10^8 CFU/mL *E. coli* HB101 as donor and *E. coli* NK5449 as recipient, which were mixed at a volume ratio of 1:1 and incubated at 37°C for 18 h. The different small letters represented significant difference among the different treatments added with PA at different amounts (Duncan's multiple-comparison test, $n = 3$, $P < 0.05$), and the asterisks indicated significant differences between the treatments with and without GSH addtion (independent sample *t*-test, n =3, * for $P < 0.05$, ** for $P < 0.01$).

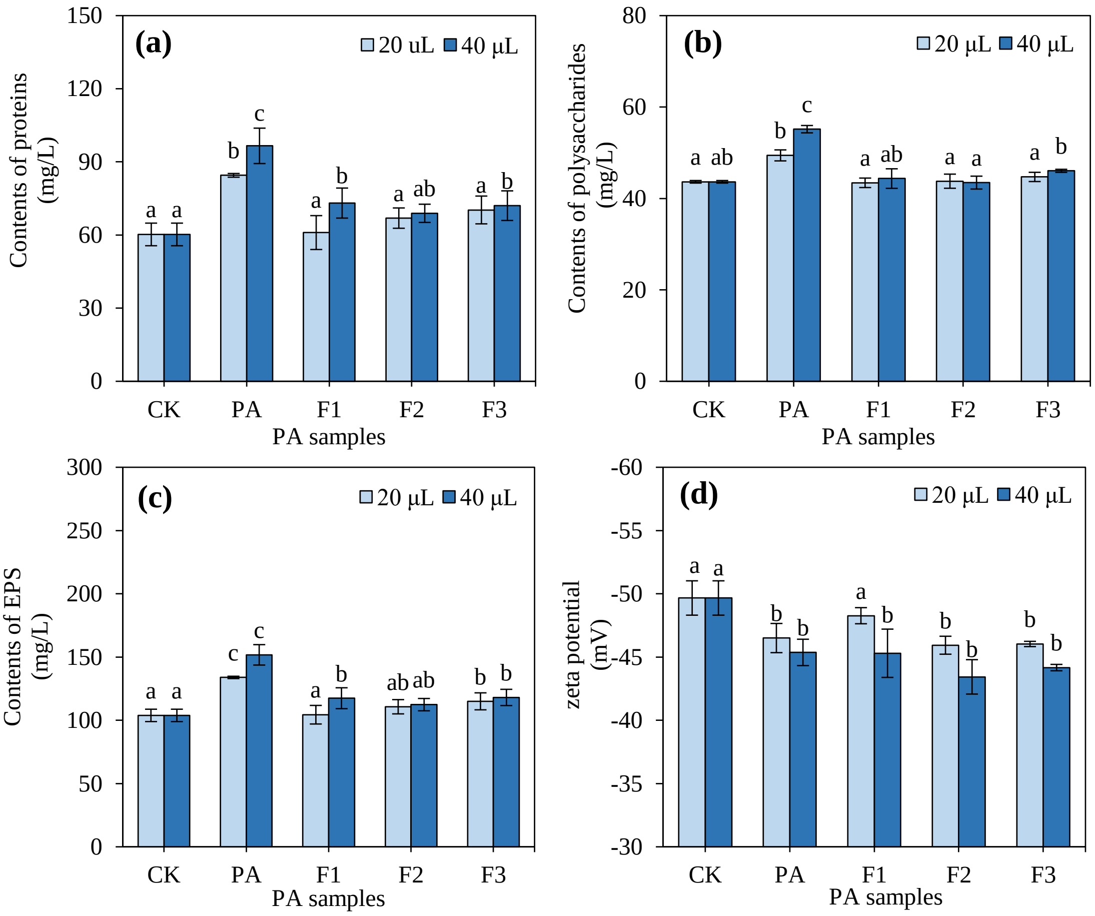

Figure S12. Effects of PA and its distilled fractions on the contents of proteins (a), polysaccharides (b), EPS (c), and the zeta potential (d) of the mixed donor *E. coli* HB101 and recipient *E. coli* NK5449. PA: the pyroligneous acid derived from blended woody waste collected from furniture factory at 450°C for 6 h. The culture conditions: 10^8 CFU/mL *E. coli* HB101 as donor and *E. coli* NK5449 as recipient, which were mixed at a volume ratio of 1:1 and incubated at 37°C for 18 h. The different small letters represented significant difference among the different treatments added with PA at different amounts (Duncan's multiple-comparison test, $n = 3$, $P < 0.05$).

Reference

1. Yu, Z.; Wang, Y.; Lu, J.; Bond, P.; Guo, J. Nonnutritive sweeteners can promote the dissemination of antibiotic resistance through conjugative gene transfer. *ISME J.* **2021**, 15, 2117–2130.

2. Lu, J.; Wang, Y.; Jin, M.; Yuan, Z.; Bond, P.; Guo, J. Both silver ions and silver nanoparticles facilitate horizontal transfer of plasmid-mediated antibiotic resistance genes. *Water Res.* **2019**, 169, 115229.

3. Li, G.; Chen, X.; Yin, H.; Wang, W.; Wong, P.; An, T. Natural sphalerite nanoparticles can accelerate horizontal transfer of plasmid-mediated antibiotic-resistance genes. *Environ. Int.* **2020**, 136, 105497.

4. Li, H.; Kang, Z.; Jiang, E.; Song, R.; Zhang, Y.; Qu, G.; Wang, T.; Jia, H.; Zhu, L. Plasma induced efficient removal of antibiotic-resistant *Escherichia coli* and antibiotic resistance genes, and inhibition of gene transfer by conjugation. *J. Hazard. Mater.* **2021**, 419, 126465.

5. Lopatkin, A.; Bening, S.; Manson, A.; Stokes, J.; Kohanski, M.; Badran, A.; Earl, A.; Cheney, N.; Yang, J.; Collins, J. Clinically relevant mutations in core metabolic genes confer antibiotic resistance. *Science* **2021**, 371 (6531), 3.

6. Kahlmeter, G.; Brown, D.; Goldstein, F.; MacGowan, A.; Mouton, J.; Österlund, A.; Rodloff, A.; Steinbakk, M.; Urbaskova, P.; Vatopoulos, A. European harmonization of MIC breakpoints for antimicrobial susceptibility testing of bacteria. *J. Antimicrob. Chemoth.* **2003**, 52 (2), 145–148.

7. Chen, S.; Li, X.; Sun, G.; Zhang, Y.; Su, J.; Ye, J. Heavy metal induced antibiotic resistance in bacterium LSJC7. *Int. J. Mol. Sci.* **2015**, 16 (10), 23390–23404.

8. Cen, T.; Zhang, X; Xie, S.; Li, D. Preservatives accelerate the horizontal transfer of plasmid-mediated antimicrobial resistance genes via differential mechanisms. *Environ. Int.* **2020**, 138, 105544.

9. Yu, K.; Chen, F.; Yue, L.; Luo, Y.; Wang, Z.; Xing, B. CeO_2 nanoparticles regulate the propagation of antibiotic resistance genes by altering cellular contact and plasmid transfer. *Environ. Sci. Technol.* **2020**, 54 (16), 10012–10021.

10. Flemming, H.; Wingender, J. The biofilm matrix. *Nat. Rev. Microbiol.* **2010**, 8 (9), 623–633.

11. Liao, J.; Huang, H.; Chen, Y. CO_2 promotes the conjugative transfer of multiresistance genes by facilitating cellular contact and plasmid transfer. *Environ. Int.* **2019**, 129, 333–342.

12. van Elsas, J.; Chiurazzi, M.; Mallon, C.; Elhottova, D.; Kristufek, V.; Salles, J. Microbial diversity determines the invasion of soil by a bacterial pathogen. *Proc. Natl. Acad. Sci. USA.* **2012**, 109 (4), 1159–1164.
13. Chen, Q.; An, X.; Li, H.; Zhu, Y.; Su, J.; Cui, L. Do manure-borne or indigenous soil microorganisms influence the spread of antibiotic resistance genes in manured soil? *Soil Biol. Biochem*. **2017**, 114, 229–237.
14. Liu, X.; Cui, R.; Shi, J.; Jiang, Q.; Gao, J.; Wang, Z.; Li, X. Separation and microencapsulation of antibacterial compounds from wood vinegar. *Process Biochem.* **2021**, 110, 275–281.
15. Korkalo, P.; Hagner, M.; Jänis, J.; Mäkinen, M.; Kaseva, J.; Lassi, U.; Rasa, K.; Jyske, T. Pyroligneous acids of differently pretreated hybrid aspen biomass: herbicide and fungicide performance. *Front Chem,* **2021**, 9, 821806.
16. Guo, X.; Zheng, P.; Zou, X.; Chen, X.; Zhang, Q. Influence of pyroligneous acid on fermentation parameters, CO_2 production and bacterial communities of rice straw and stylo silage. *Front Microbiol.* **2021**, 12, 701434.
17. Zheng, H.; Wang, R.; Zhang, Q.; Zhao, J.; Li, F.; Luo, X.; Xing, B. Pyroligneous acid mitigated dissemination of antibiotic resistance genes in soil. *Environ. Int.* **2020**, 145, 106158.
18. Zhang, Y.; Wang, X.; Liu, B.; Liu, Q.; Zheng, H.; You, X.; Sun, K.; Luo, X.; Li, F. Comparative study of individual and co-application of biochar and wood vinegar on blueberry fruit yield and nutritional quality. *Chemosphere* **2020**, 246, 125699.
